# Labyrinthine Microstructures with a High Dipole Moment Boron Complex for Molecular Physically Unclonable Functions

**DOI:** 10.1021/acsami.5c13228

**Published:** 2025-10-29

**Authors:** Tevhide Ayça Yıldız, N. Burak Kiremitler, Nilgun Kayaci, Mustafa Kalay, Emrah Özcan, İbrahim Deneme, Zehra Coşkun, Mustafa Serdar Onses, Bünyemin Çoşut, Hakan Usta

**Affiliations:** † Department of Materials Science and Nanotechnology Engineering, 346448 Abdullah Gül University, Kayseri 38080, Türkiye; ‡ ERNAM - Nanotechnology Research and Application Center, 52958 Erciyes University, Kayseri 38039, Türkiye; § Department of Materials Science and Engineering, Erciyes University, Kayseri 38039, Türkiye; ∥ Department of Electricity and Energy, Kayseri University, Kayseri 38039, Türkiye; ⊥ Department of Chemistry, Faculty of Science, Gebze Technical University, Gebze, Kocaeli 41400, Türkiye; # Department of Physics, Faculty of Science, University of South Bohemia, Branišovská 1760, České Budějovice 370 05, Czech Republic

**Keywords:** physically unclonable functions, anticounterfeiting, labyrinthine microstructure, dipolar boron complex, high dipole moment

## Abstract

The design and development of novel *molecular*-physically unclonable functions (PUFs) with advanced encoding characteristics and ease of fabrication have recently attracted attention in cryptography, secure authentication, and anticounterfeiting. Here, we report the development of a new high dipole-moment small molecule, InIm-BF_2_, a difluoroborate complex of an indolyl-imine ligand, and the fabrication of unique labyrinthine patterns through a facile two-step thin film process under ambient conditions. The new molecule has a dipolar, coplanar π-backbone and arranges in the solid state with antisymmetric cofacial π-stackings (3.86 Å). These properties, along with short C–H···π contacts (2.74–2.88 Å) and nonclassical C–H···F hydrogen bonds (2.47–2.51 Å) (23.4% and 11.5% of the Hirshfeld surfaces, respectively), drive the formation of amorphous *molecular* PUF patterns with disordered, short-range interactions. Spin-coating followed by thermal annealing at a moderate temperature produces nanoscopic molecular thin films with intricate labyrinthine patterns. These patterns, characterized by interconnected, irregularly shaped, micron-sized (≈50–100 μm) features, exhibit excellent PUF characteristics, verified through advanced image analysis and computational algorithms. Unlike randomly positioned isolated features in classical binarized keys, the interconnected labyrinthine patterns possess rich entropy and complex features, directly authenticated via deep-learning methodologies. Our work not only demonstrates a facile, promising approach to fabricating unique high-entropy PUF patterns but also provides critical insights into designing advanced molecular materials for next-generation security applications.

## Graphical abstract

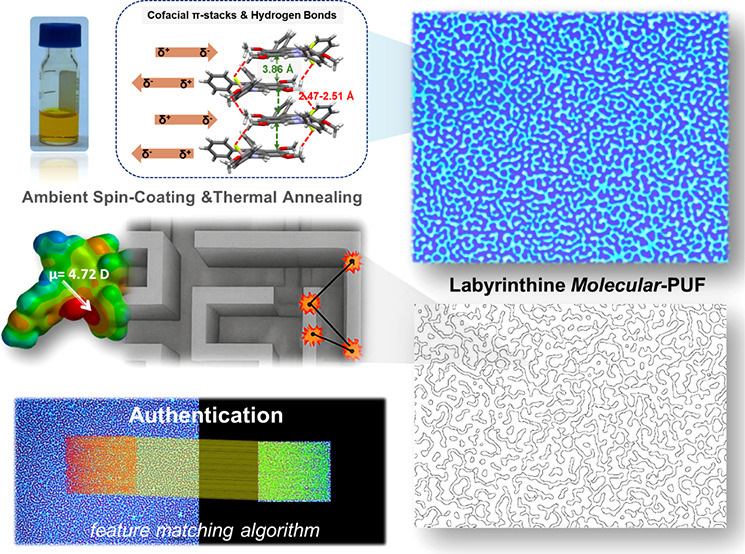

## 1 Introduction

The synthesis of novel organic structures and the development of their functional thin films are becoming increasingly critical for advancing high-tech applications.^1^ Recently, physically unclonable functions (PUFs) have emerged as a promising application of organics in realizing a high level of security across diverse fields, from data protection to anticounterfeiting of commercial goods in global market.^2–4^ PUFs were first introduced by Pappu et al. in 2002 as physical one-way functions based on optical scattering within the disordered medium of inhomogeneous epoxy-based structures.^5^ Compared to mathematical one-way functions, PUFs provide significant advantages, such as unclonability due to intrinsic physical randomness (i.e., absence of a deterministic process), exceptional capability for encryption key generation and authentication, and ease of fabrication and miniaturization, making them virtually impervious to third-party attacks.^5–7^ Over the years, the emergence and success of PUFs have undoubtedly been driven by the diversity of materials and the structural engineering at both nano- and microscales utilized in these systems. These materials include plasmonic nanoparticles,^8^ two-dimensional materials,^9^ edible biocompatible materials,^10^ polymers,^2,11^ organic semiconductors,^12^ light-emitting molecules,^6,13^ and structured thin films.^5,14,15^ Among these, nanoscopic films (thickness <100 nm) composed of organic materials are particularly interesting due to the wide range of supramolecular features they can potentially exhibit. These features arise from inherently random physical processes that can be manipulated to achieve desired morphological patterns. For instance, processes such as thermal- and solvent-annealing induced dewetting of a continuous nanoscopic organic film result in the spontaneous formation of randomly positioned and oriented domains. Recently, our research groups have successfully demonstrated the promise of this facile approach using both polymeric and molecular nanoscopic films for PUF applications.^13,14^ Compared to other materials, organic structures offer virtually unlimited possibilities through exploratory synthesis, enabling unique, proprietary designs for specific companies and applications.^16^ PUF fabrication with organics could be both straightforward and adaptable for mass production, utilizing various low-cost deposition methods (e.g., physical vapor deposition, drop-casting, spin-coating) and processing techniques (e.g., thermal and solvent vapor annealing).^13,17,18^ Additionally, organic materials are compatible with various flexible substrates, such as plastics and paper, and can be coated over large areas.^19,20^ Therefore, designing and developing novel organic structures and harnessing their unique properties are crucial for expanding the diversity and functionality of PUFs.

Among the diverse classes of organic materials, small molecules offer monodispersity and high synthetic reproducibility, along with key advantages such as ease of purification, ultrahigh chemical purity, and good solubility in common solvents.^6,13^ Furthermore, molecular-level structural engineering enables fine-tuning of optoelectronic and physicochemical properties, as well as control over microstructure and morphology.^21^ Tunable noncovalent interactions and molecular self-assembly provide a distinct advantage for their use in PUFs, enabling the realization of diverse features by employing solution- and thermal-based processing.^22–24^ These characteristics can contribute significantly to the diversity and functionality of PUFs. The molecular properties for PUFs stem directly from their chemical structures, which encompass distinct chemical bonds and atomic arrangements. To this end, molecular structures with heteroatoms—particularly boron-containing organic small molecules—are intriguing, as they can generate strong local bond dipoles and molecular dipoles, thereby inducing diverse van der Waals interactions that can give rise to unique PUF patterns.^25^

Boron-containing small organic molecules, namely organoboranes, have gained increasing popularity in the last few decades based on their unique optoelectronic properties and tunable crystallinity. Building on the pioneering reagent-based synthesis studies that led to the Nobel Prize in Chemistry in 1979, organoboranes have become central in a wide range of academic and industrial applications.^26^ Their structural versatility, enabling diverse functionalization and substitutions, has driven the development of a wide array of boron-containing organic materials.^27^ Introducing boron into π-conjugated backbones is an effective and practical strategy for developing novel materials with enhanced thin-film and crystallinity properties.^28^ Among varied organoboron compound families, difluoroborate­(BF_2_) complexes are attractive small molecules, exhibiting high photoluminescence quantum yields and excellent photophysical properties.^29^ The difluoroborate ring in complex molecules shows structural rigidity with π-delocalization and a large dipole moment (μ > 3–4 D), which is key to tunable noncovalent interactions for molecular self-assembly.^30,31^ In the last few decades, small molecules with a BF_2_-complex structure have found extensive applications in diverse fields, including biological labeling,^32^ surface-enhanced Raman scattering,^33^ photovoltaics,^34^ photodynamic therapy,^35^ biomedicine and biological imaging.^36^ Consequently, research interest in the development of bidentate N-donor ligand systems and the synthesis of their novel complexes with BF_2_ persists to this day.^37,38^ However, to the best of our knowledge, PUF studies involving organoborane molecules have not yet been reported in the literature.

In this study, we present the design, synthesis, and characterization of a new *molecular* PUF material **InIm-BF**_**2**_, which is the difluoroborate­(BF_2_) complex of an indolyl-imine ligand system (Figure 1a). This molecule exhibits an exceptionally large dipole moment (μ = 4.72 D), originating from the dimethoxypart and oriented toward the 4,4-fluorine substituents (Figure 1a). On the basis of single crystal characterizations, C–H···π and C–H···F short contacts and π···π interactions between antisymmetrically arranged dipolar difluoroborate backbones were observed. These intermolecular interactions were found to be key in the formation of interconnected and irregularly shaped labyrinthine PUF patterns. Unlike our earlier *polymeric*- and *molecular*-PUFs,^13,14^ the spin-coated nanoscopic (≈25 nm) thin film of **InIm-BF**_**2**_ does not exhibit complete dewetting after thermal annealing at moderate temperatures. Instead, interconnected molecular domains were observed as a result of the presence of short-range intermolecular interactions during self-assembly under thermal annealing conditions. These patterns, characterized by interconnected, irregularly shaped, micron-sized (≈50–100 μm) features, exhibit excellent PUF characteristics as verified through advanced image analysis and computational algorithms. They can be directly authenticated via deep-learning methodologies. The interconnectivity and the formation of complex, continuous networks on substrate surface present a promising approach to enhance entropy, moving beyond the conventional reliance on domain-based binary encoding (Figure 1b). Encoding strategies based on interconnected domains have recently been demonstrated on a limited range of surfaces,^2,4,39–47^ including polymeric particles with randomly generated silica film wrinkles^4^ and a light-controlled supramolecular network formed by a copolymer and a small molecule.^44^ In addition to the optical microscope images recorded for the present *molecular*-PUFs, the inherent light-emitting and Raman-scattering characteristics of **InIm-BF**_**2**_ could enable luminescent PUFs with enhanced multiplex encoding. Considering the laborious and complex fabrication and readout processes of PUFs in the literature,^40^ our current *molecular*-PUF system shows a promising and facile approach.

**Figure 1:**
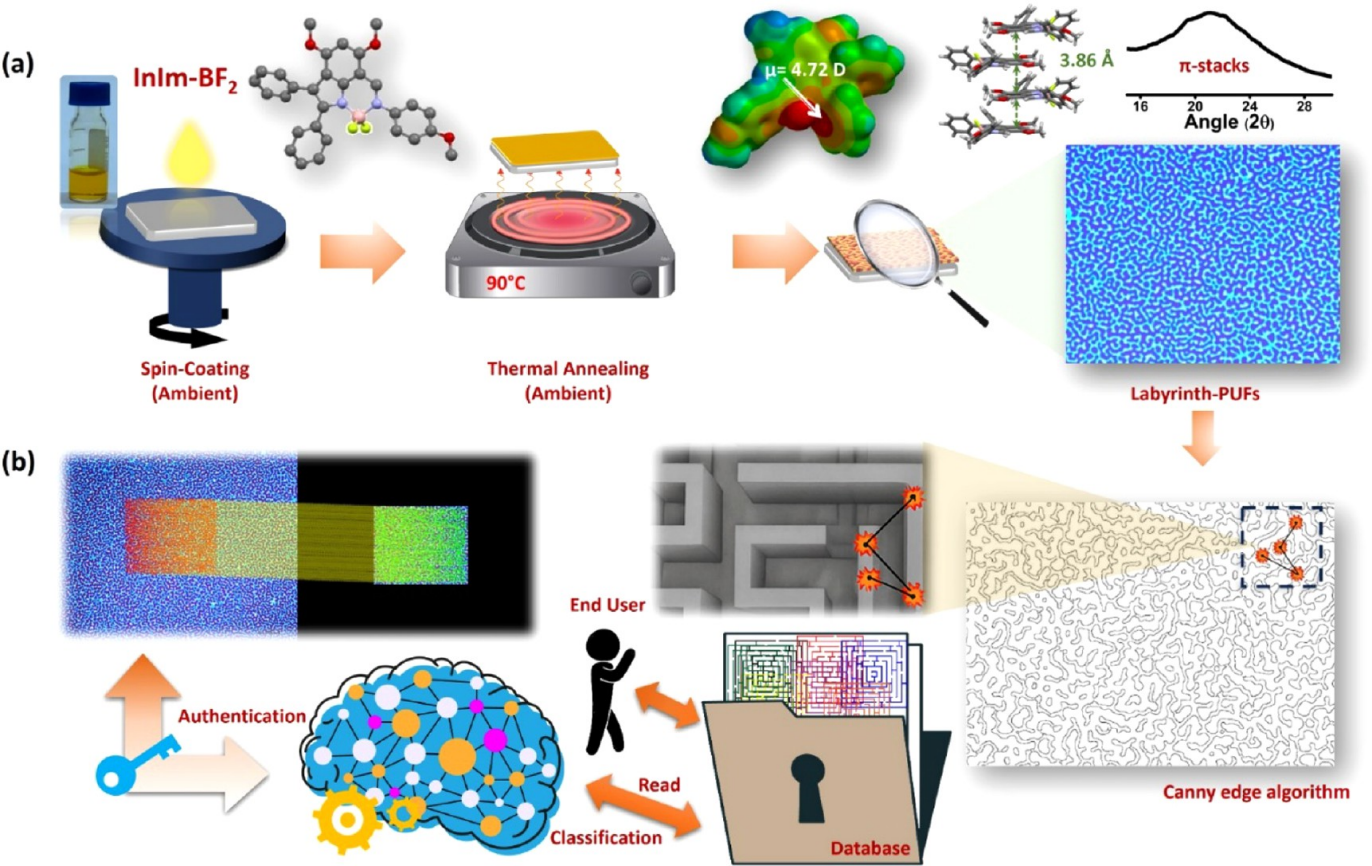
(a) Fabrication steps of *molecular*-PUFs having labyrinthine patterns, which involve spin-coating of an **InIm-BF**_**2**_ solution in chloroform onto a silicon substrate, followed by thermal annealing on a hot plate under ambient atmosphere. Electrostatic potential surface (EPS) map, molecular dipole moment, and cofacial π-stacksof **InIm-BF**_**2**_ molecules, along with the XRD pattern and the optical microscope imaging of *molecular*-PUFs. (b) Determination of features on the labyrinthine patterns as a result of Canny and Jaccard algorithms and illustration of authentication procedure.

## 2 Experimental Section

### 2.1 Materials and Methods

All reagents, used without further purification, were purchased from Sigma-Aldrich (USA), and all solvents were obtained from Merck (USA). Reactions were monitored by thin-layer chromatography (TLC) using Merck TLC Silica gel 60 F254. Silica gel column chromatography was performed using Merck Silica gel 60 (particle size: 0.040–0.063 mm, 230–400 mesh ASTM). ^1^H NMR and ^13^C NMR spectra for all compounds were recorded in CDCl_3_ using a Varian INOVA 500 MHz spectrometer. Electrothermal IA 9000 Series melting point apparatus was used for the melting temperature measurement. Differential scanning calorimetry (DSC) and thermogravimetric analysis (TGA) were performed on Mettler Toledo DSC822e and TGA/SDTA851e instruments, respectively. Indium and zinc standards (Mettler Toledo, Schwerzenbach, Switzerland) were used for calibration in DSC. UV–vis absorption spectra were recorded using a Shimadzu 2101 UV–vis spectrophotometer. Fluorescence excitation and emission spectra were measured on a Varian Eclipse spectrofluorometer with 1 cm path length cuvettes at room temperature. Fluorescence lifetimes were determined using a Horiba Jobin-Yvon SPEX Fluorolog 3–2iHR instrument equipped with a Fluoro Hub-B Single Photon Counting Controller, with an excitation wavelength of 360 nm for all compounds. Signal acquisition was carried out using a time-correlated single photon counting (TCSPC) module.

### 2.2 Synthesis, Structural Characterization, and Single-Crystal Study

The synthesis of 4,6-dimethoxy-2,3-diphenyl-1H-indole-7-carbaldehyde (**1**) was conducted in two steps in accordance with our previously reported procedures.^48^ X-ray data collection and structure refinement details for **InIm-BF**_**2**_ *single crystal* are provided in the ().

#### 2.2.1 Synthesis of InIm

To a stirred solution of 4,6-dimethoxy-2,3-diphenyl-1H-indole-7-carbaldehyde (**1**) (100 mg; 0.280 mmol) in ethanol (20 mL), 4-methoxyaniline (51 mg; 0.41 mmol) and glacial acetic acid (50 μL) were added, and the mixture was stirred under reflux for 2 days. The solvent was removed under reduced pressure and the crude product was washed/rinsed with ethanol (50 mL). The resulting orange solid was filtered to give the **lnlm** solid (92 mg) in 72% yield. ^1^H NMR (; 500 MHz, CDCl_3_) δ 12.15 (s, 1H, NH), 10.55 (s, 1H, CH=N),7.95–7.21 (m, 14H, phenyls), 6.31 (s, 1H, indole-H), 4.11, 3.73, and 2.89 ppm (s, 9H, O–CH_3_) ppm; ^13^C NMR (; 125 MHz, CDCl_3_) δ 156.26, 155.15, 150.48, 142.76, 135.95, 132.97, 132.42, 131.45, 128.33, 128.08, 127.26, 126.88, 125.84, 122.08, 121.79, 118.54, 110.01, 87.73 (ArC), 56.74, 55.21 ppm (O–CH_3_). MALDI TOF (*m*/*z*) () C_30_H_26_N_2_O_3_ [M]^+^ calculated 462.19, found: 445.21 [M-OCH_3_] ^+^ *m*/*z*.

#### 2.2.2 Synthesis of InIm-BF_2_

Triethylamine (1 mL) and BF_3_·Et_2_O (1.5 mL) were added to a stirred solution of **InIm** (100 mg; 0.216 mmol) in toluene (35 mL). The reaction mixture was heated to reflux and stirred for 1 day. The resulting mixture was concentrated under reduced pressure and purified by flash column chromatography (silica gel, dichloromethane: hexane; 3:1 (v/v)) to yield ***InIm-BF***_***2***_as an orange powder (70.0 mg, 64%). ^1^H NMR (; 500 MHz, CDCl_3_) δ 8.67 (s, 1H, CH=N), 7.45–6.90 (m, 14H, Aryl-CH), 6.15 (s, 1H, Aryl-CH), 4.00 (s, 3H, OCH_3_), 3.91 (s, 3H, OCH_3_), 3.81 (s, 3H, OCH_3_) ppm; ^13^C NMR (; 125 MHz, CDCl_3_) δ 164.02, 161.31, 158.96, 154.25, 139.46, 138.44, 137.35, 134,85, 132.81, 131.04, 130.98, 127.17, 127.06, 126.82, 125.55, 125.37, 113.97, 86.58 (Aryl-C), 55.90, 55.28 (OCH_3_), ppm; MALDI TOF (*m*/*z*) () C_30_H_25_BF_2_N_2_O_3_ [M^+^] calculated 510.19, found: 511.19 [M]^+^, 491.29 [M-F]^+^ *m*/*z*.

### 2.3 Solution-Based Fabrication and Characterization of *Molecular*-PUFs

The substrate used for PUF fabrications is a heavily *p*-doped silicon substrate/wafer having a thermally grown 300 nm thick SiO_2_ layer. The substrates were subjected to cleaning in an ultrasonic bath using hexane, acetone and ethanol, respectively (10 min for each solvent). Subsequently, UV-Ozone treatment (UV/Ozone ProCleaner Plus) was conducted for a duration of 25 min to activate the substrate surface. The molecular thin films were deposited directly onto the cleaned substrates by spin-coating the corresponding **InIm-BF**_**2**_ solution in CHCl_3_ (Sigma-Aldrich) at concentrations of 2.0 mg/mL, 4.0 and 8.0 mg/mL, at 1100 rpm under ambient conditions. After the spin-coating process, the thin films were thermally annealed by heating the substrates on a hot plate at temperatures of 90 °C, 100 °C, and 110 °C for 30, 60, 75, and 120 min under ambient conditions. Optical images were recorded using a ZEISS Axio Imager 2 microscope. The surface morphologies and topography of the thin films were investigated via atomic force microscopy (AFM) on NanoSurf, FlexAFM C3000 and scanning electron microscope (SEM) on Zeiss-Gemini SEM 300. X-ray diffraction (XRD) measurements were conducted on an X-ray diffractometer (Bruker AXS D8).

### 2.4 Analysis and Authentication of PUFs

The analysis, processing, matching and authentication of images were performed in a MATLAB computing platform. Conventional PUF analysis involved binarization of images to generate binary keys and calculation of several metrics based on these keys. Details of such conventional analysis are given in the .

#### 2.4.1 Detection of Edges and Jaccard Similarity

To authenticate the labyrinth-like molecular PUFs, edge points of the features were first detected using the Canny Edge Algorithm.^49,50^ This algorithm detects sharp transitions and identifies edges where these transitions are dense in an image. During the image processing phase, the labyrinth pattern image was first converted to grayscale, followed by the application of Canny edge detection. Canny edge points were determined for each image.^51^ The Jaccard similarity matrix was calculated to compare the similarity between these images. Jaccard similarity is a method to measure the similarity between two clusters based on the ratio of the size of the intersection set (common elements) to the size of the union set (all elements).^52^ This similarity is mathematically defined by using eq 1, where A and B refer to two sets of data.

1 $$J\left(A,B\right)=\frac{\left|A\cap B\right|}{\left|A\cup B\right|}$$

The Jaccard similarity matrix was calculated for 30 images to compare the similarity of these images with each other.

#### 2.4.2 Authentication Process

In this study, a feature detection-based algorithm Oriented FAST and Rotated BRIEF (ORB) was used for feature matching of labyrinthine patterns. The ORB key point detection algorithm, which identifies and stores key points in each image as objects, was used. A deep convolutional neural network (CNN)-based methodology was developed for the authentication of labyrinthine features. A deep CNN with a ResNet-50 architecture was implemented in the MATLAB environment for pattern recognition. Eighty percent of the data set was allocated for training, and 20% for validation. The bag-of-features (BoF) technique was used for feature extraction from the training data. In total, the data set consists of 1000 images taken under various scaling, brightness, and rotation conditions.

## 3 Results and Discussion

### 3.1 Synthesis and Structural/Physicochemical Characterizations

The synthesis of the new molecule, **InIm-BF**_**2**_, is outlined in Figure 2a. The synthesis begins with the preparation of the bidentate indolyl-imine ligand, **InIm**, by reacting 4,6-dimethoxy-2,3-diphenyl-1H-indole-7-carbaldehyde (**1**) with electron-rich 4-methoxyaniline in ethanol, using a catalytic amount of glacial acetic acid (72% yield). This Schiff base reaction proved to be an efficient method for obtaining the present ligand. It is noteworthy that compound **1** was prepared in two in steps in accordance with our earlier report.^48^ In the synthesis of **1**, 3,5-dimethoxyaniline reacts with benzoin in the presence of aniline in acetic acid at 130 °C, yielding 2,3-diphenyl-4,6-dimethoxyindole, which then serves as the starting material for a Vilsmeier–Haack reaction to yield **1**. Since plain indole does not react at the C7 position, the 2,3-diphenyl and 4,6-dimethoxy substitutions enable selective formylation at this site, forming the foundation of our synthetic strategy. The indolyl-imine ligand, **InIm**, was complexed with boron trifluoride in toluene using triethylamine as a base, yielding **InIm-BF**_**2**_ in 64% yield. The chemical structure and the purity of the solids were confirmed by ^1^H and ^13^C NMR spectroscopy () and MALDI-TOF mass spectrometry (). Notably, the indole (−NH) proton of the **InIm** ligand disappeared in **InIm-BF**_**2**_, consistent with successful complexation with difluoroborate. The molecular structure of **InIm-BF**_**2**_, having a six-membered difluoroborate ring, was further confirmed through single-crystal X-ray diffraction analysis (see for further details, Figure 2b and ). Thermogravimetric analysis (TGA) showed a good thermal stability with a thermolysis onset temperature of >300 °C. Differential scanning calorimetry (DSC) revealed a sharp endothermic peak at 289 °C, corresponding to the crystalline-to-isotropic liquid transition, which is consistent with the measured melting temperature (290–291 °C) of the solid (). The photophysical properties of **InIm-BF**_**2**_ were investigated in toluene at room temperature using steady-state absorption and fluorescence spectroscopy (). The absorption spectrum exhibits two distinct peaks, a high-energy transition at 386 nm and a lower-energy shoulder near 420 nm. The fluorescence spectrum reveals a strongly red-shifted emission with a maximum at 580 nm, corresponding to a large Stokes shift of ∼200 nm. This suggests a notable difference between ground- and excited-state dipole moments. Fluorescence lifetime measurements support the observed large Stokes shift, yielding a relatively long lifetime of 5.12 ns ().

**Figure 2:**
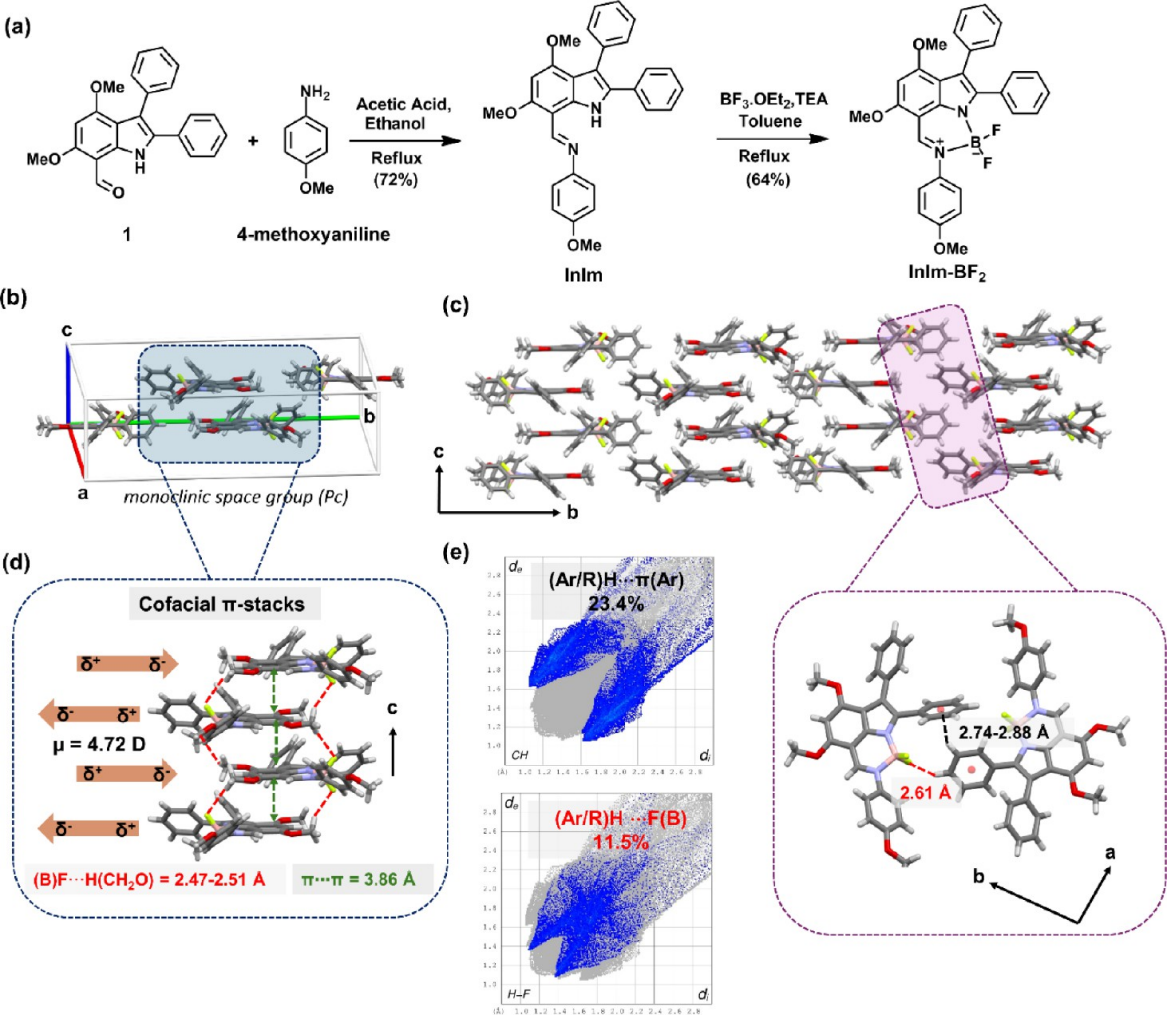
(a) Synthesis of **InIm-BF**_**2**_. (b) Unit cell for the single-crystal of **InIm-BF**_**2**_ and the corresponding coplanar molecular structures (with tilted peripheral phenyls) and the space group. (c) Molecular packing along the crystallographic *bc*-plane. (d) Cofacial π-stacks of **InIm-BF**_**2**_ molecules along the crystallographic *c*-axis, the antiparallel arrangement of molecular dipoles, and key intermolecular interactions (π···π (between dimethoxybenzenes) and (OCH_2_)­H···F­(B) hydrogen bonds). (e) Hirshfeld surfaces of **InIm-BF**_**2**_ mapped with d_norm_ representing two major intermolecular interactions and the illustration of the key intermolecular interactions ((Ar/R)­H···F­(B) and (Ar/R)­H···π­(Ar)) along the crystallographic *ab*-plane.

### 3.2 Single Crystal Characterization and Intermolecular Interactions

The new molecule, **InIm-BF**_**2**_, was studied using single crystal X-ray diffraction to gain a comprehensive understanding of its molecular structure, packing properties, and intermolecular interactions. Details of the X-ray data collection and structure refinement are provided in the (). As shown in Figure 2b, **InIm-BF**_**2**_ crystallizes in the monoclinic space group (*P*c), and the π-conjugated backbone of its central indolyl-iminedifluoroborate core (C_9_BN_2_) exhibits a nearly coplanar structure. The peripheral phenyl and 4-methoxyphenyl groups adopt twisted conformations with respect to the central indolyl-imine difluoroborate π-core, showing dihedral angles of 42.75–47.52°. Along the crystallographic *bc*-plane (Figure 2c), cofacial π-stacks of **InIm-BF**_**2**_ molecules were observed. The π–π interactions occur along the crystallographic *c*-axis in a zigzag motif, forming one-dimensional molecular stacks. Short interplanar π–π distance of 3.86 Å was measured between dimethoxybenzene rings of the indolyl-imine difluoroborate cores. Most importantly, the molecular dipoles adopt an antiparallel arrangement, with the slippage of about one phenyl ring repeating in alternating opposite directions (Figure 2d). This slippage enables the formation of short (OCH_2_)­H···F­(B) (2.47–2.51 Å < r_vdw_(F) + r_vdw_(H) = 2.67 Å) hydrogen bonds among the adjacent molecular layers. The presence of a strong molecular dipole moment (μ = 4.72 D) on the indolyl-imine difluoroborate π-core enables energetically favorable antiparallel dipole–dipole interactions.^53,54^ On the other hand, along the crystallographic *ab*-plane (Figure 2e), the molecules forming the one-dimensional cofacial π-arrangements interact with each other via strong (Ph)­H···F­(B) (2.61 Å < r_vdw_(F) + r_vdw_(H) = 2.67 Å) and (Ph)­H···π­(Ph) (2.74–2.88 Å < r_vdw_(C) + r_vdw_(H) = 2.90 Å) contacts. Additional (Ar)­H···π­(Ar) interactions (2.77–2.82 Å) were observed between the methoxyphenyl and phenyl (i.e., -*meta* to the indole nitrogen) units as well, along the crystallographic *a*-axis. All these interactions enable **InIm-BF**_**2**_ molecules to form the 3D self-assembly. Hirshfeld surface analysis of **InIm-BF**_**2**_ crystal lattice revealed that (Ar/R)­H···F­(B) and (Ar/R)­H···π­(Ar) interactions constitute 11.5% and 23.4% of the total Hirshfeld surface, respectively.

### 3.3 *Molecular*-PUF Fabrication and Characterizations

Figure 1a illustrates the facile fabrications steps for our *molecular*-PUFs. These steps include spin-coating of an **InIm-BF**_**2**_ solution in chloroform onto a silicon substrate (with a thermally grown 300 nm thick SiO_2_ layer) to form a nanoscopic thin-film, followed by thermal annealing on a hot plate. Both steps were performed under ambient conditions. To study the dewetting behavior of the molecular thin film and achieve the desired interconnected PUF patterns, we optimized key parameters such as solution concentration, annealing temperature, and annealing time. A modest annealing temperature of 90 °C for 60 min was initially tested using three different spin-coating concentrations: 2.0 mg/mL, 4.0 mg/mL, and 8.0 mg/mL (Figure 3a). As the spin-coating solution concentration increased, the thickness of the **InIm-BF**_**2**_ thin film increased from 10 nm at 2.0 mg/mL to 25 nm at 4.0 mg/mL and 45 nm at 8.0 mg/mL. While the 2 mg/mL solution resulted incompletely dewetted surface (Figure 3a, left image), the 8 mg/mL solution showed poor dewetting behavior (Figure 3a, right image). The 4 mg/mL solution concentration was found to be ideal for forming randomly oriented, interconnected features, highlighting the critical role of achieving an optimal film thickness. This observation is consistent with our earlier findings on other molecular thin films.^13^ After observing that thin films prepared from the 4 mg/mL solution and annealed at 90 °C exhibited ideal behavior for labyrinthine PUF formation, slightly higher temperatures of 100 and 110 °C were explored (Figure 3b). Interestingly, at these higher temperatures, much larger, polygonal hole formations were observed, while the molecular domains remained interconnected around the holes. The elevated temperatures apparently enhance molecular diffusion, leading to the formation of micron-sized polygon-like holes and arranging the molecular features around these holes into a cellular pattern.^55^ Similar cellular pattern structures can be found in nature in plants, soap froths, and desert soils. As the final step of optimization, shorter (30 min) and longer (75 and 120 min) annealing times were tested using the optimal solution concentration (4.0 mg/mL) and annealing temperature (90 °C). While the shorter annealing time (30 min) resulted in only nucleation for hole formation without producing the desired interconnected features, the longer annealing time of 120 min yielded again micron-sized, polygon-like holes as a result of enhanced molecular diffusion. The desired labyrinthine PUF patterns were achieved at both 60 and 75 min, with no discernible differences observed between the two.

**Figure 3:**
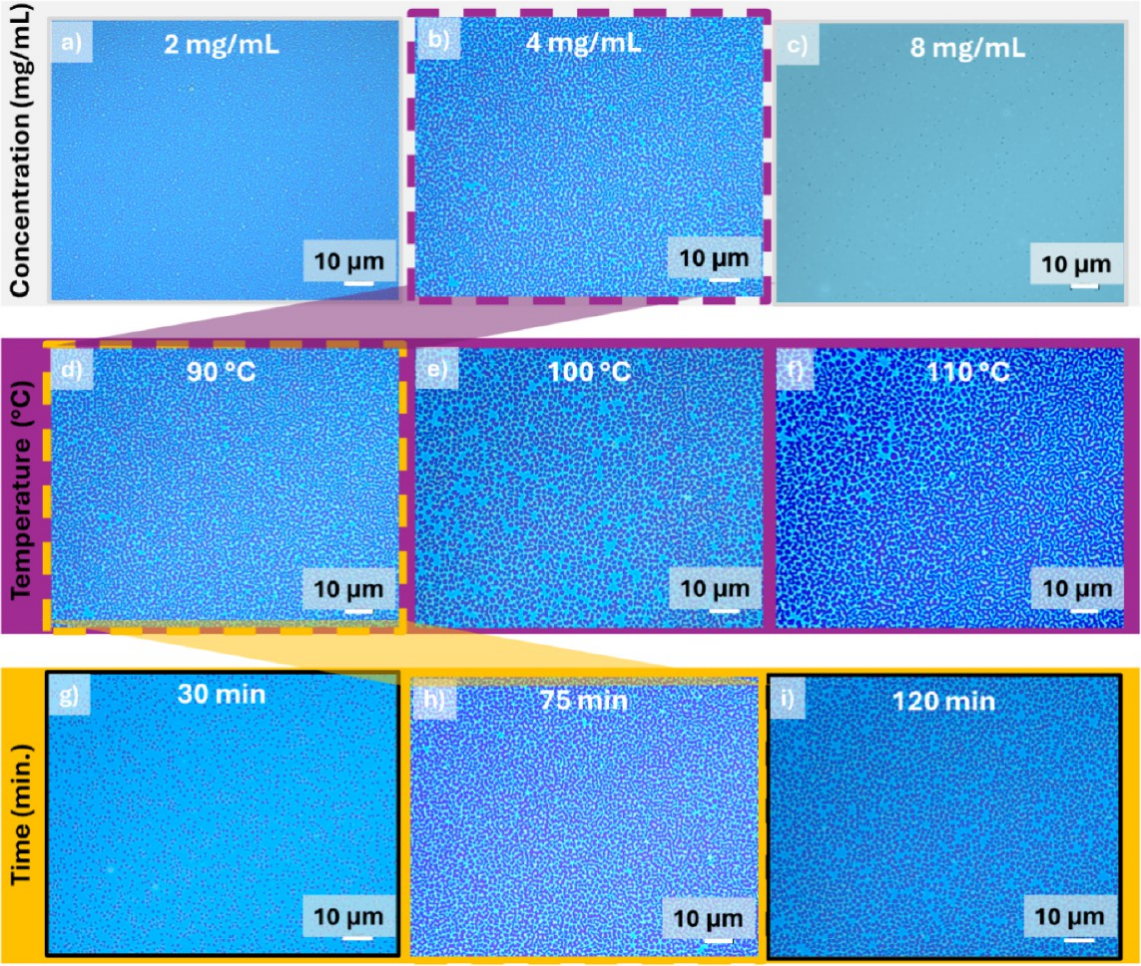
Optical images obtained for the *molecular*-PUF patterns for varied spin-coating solution concentrations (a–c) with a thermal annealing at 90 °C for 60 min, for varied annealing temperatures (d–f) performed for 60 min and starting with 4 mg/mL spin-coating concentration, and for varied thermal annealing times (g–i) for 4 mg/mL spin-coating concentration and 90 °C of annealing temperature.

The morphological and microstructural properties of the **InIm-BF**_**2**_ labyrinthine patterns were characterized using atomic force microscopy (AFM), scanning electron microscopy (SEM), and θ–2θ X-ray diffraction analysis. As shown in Figure 4b,c, uniform and continuous labyrinthine patterns were observed in both the SEM and AFM images, with interconnection lengths reaching ≈50–100 μm in some features, while the widths typically range from 0.5 to 2.0 μm. The heights of these labyrinthine patterns range from 65 to 135 nm (Figure 4c, inset), indicative of the physical randomness for molecular diffusion and deposition during the formation of these features via thermal annealing. The average height of the labyrinthine features is consistent with the initial thickness (25 nm) and the surface coverage of dewetted features. As shown in Figure 4d, the labyrinthine features lack crystallinity in the θ–2θ X-ray diffraction, displaying only a broad high-angle halo peak at 2θ from 17° to 30°. This suggests a nearly amorphous microstructure with disordered short-range intermolecular interactions.^56,57^ The broad peak, centered at 2θ = 21.83°, corresponds to a *d*-spacing of ∼4.1 Å in the out-of-plane direction. Based on single-crystal solid state analysis, this corresponds to the cofacial π–π stacks (Figure 2d, π···π ∼ 3.9 Å), stabilized by strong dipolar interactions. Additionally, considering that this broad peak extends to larger 2θ values of up to ∼30°, short-range CH···F and CH···π contacts (≈2.5–2.9 Å) likely contribute to the structural stability and integrity of the labyrinthine features.

**Figure 4:**
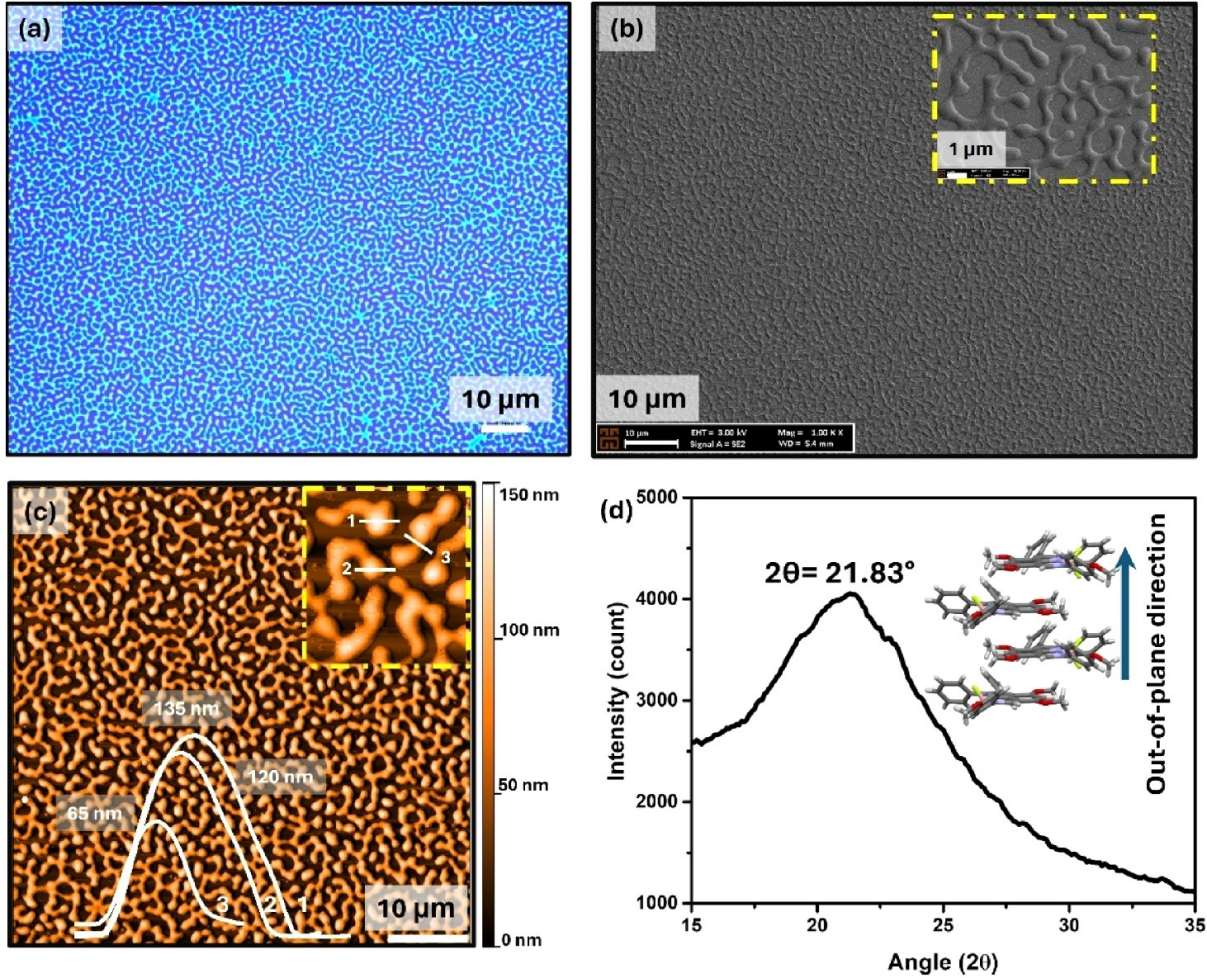
(a) The optical microscope image, (b) SEM images, (c) AFM images showing the height profiles for the zoomed-in region, and (d) θ-2θ XRD pattern, showing the broad halo peak and the illustration of π-stacks in the out-of-plane direction, for **InIm-BF**_**2**_ labyrinthine *molecular*-PUF patterns (scale bar 150 nm).

**InIm-BF**_**2,**_ due to its excellent solubility and solution processability, provides a key advantage for scalable fabrication of *molecular*-PUFs. We demonstrated that our ambient-atmosphere fabrication method, utilizing spin-coating followed by thermal annealing, is effective on a 2-in. silicon wafer () and extends to other substrates, including microscope glass and silicon with a native oxide layer (). Across the entire silicon wafer surface, uniformly distributed labyrinthine-like structural features were consistently observed. The high reproducibility of these structural patterns over large areas and across different substrates underscores the potential of this approach for large-scale PUF production.

### 3.4 PUF Analysis and Authentication

To evaluate the performance of the labyrinth pattern for PUF applications and its potential for implementation in high-security authentication, our approach is built upon a multilayered security paradigm. This paradigm integrates three distinct and comprehensive methodologies, as will be detailed throughout this section to demonstrate their individual strengths and collective synergy: binary key analysis, direct feature matching, and convolutional neural network based (CNN) classification (). This multifaceted strategy is designed to ensure robust and reliable authentication by using the unique strengths of each technique, thereby compensating for the potential limitations of any single approach.

In order to assess the randomness of microscopic features resembling labyrinths and their potential application as PUFs, we implemented several computational image analysis procedures. The most prevalent method involves the encoding of an optical image into a binary key, which is a sequence of 1-bits and 0-bits. The classical von Neumann debiasing method was employed for key extraction. This method is effective in eliminating lengthy sequences of a single type of bit, an occurrence that arises from the dominance of a single type of response in physical systems. For each of the 30 samples analyzed, this approach extracted binary keys that were 256 bits in length (). The uniformity, uniqueness, and Hamming distances were computed using these binary keys (). The ideal uniformity value is 0.5, and the distribution of 0 and 1 bits must be nearly equal for the generated keys to be considered random. The uniformity value was determined to be 0.5173, which is very close to the ideal value of 0.5. Interchip Hamming distance (*HD*_*INTER*_) values were calculated to analyze the uniqueness of generated binary keys. The uniqueness was found to be 0.5106, a value that is very close to the ideal value of 0.5. *HD*_*INTER*_ values exhibited a Gaussian distribution with a standard deviation of 0.0781. These results confirm the system’s capacity to generate security keys that are significantly unique. The average of *HD*_*INTRA*_ values was 0.0446, which approaches zero and signify reproducibility of the PUF responses under identical conditions. In addition, false positive and false negative rates were determined by taking into account interdevice and intradevice variability. With the determined cutoff threshold of 0.1298, the false positive rate was calculated as 1.52 × 10^–8^ and the false negative rate as 1.35 × 10^–1^. These findings demonstrate that the system has high reliability and low error rates.

To assess the randomness of the keys obtained from the PUFs, a series of statistical tests directed by the National Institute of Standards and Technology (NIST) were employed.^58^ Seven different tests were performed to evaluate the randomness of 7680-bit digitized keys that were extracted from 30 distinct PUFs. The results of the randomness tests, which utilize the chi-square (χ^2^) distribution test, are provided in . In order to ensure statistical accuracy in the test of *p*-value uniformity, a minimum of 55 sequences were required to be processed. NIST testing was performed to 60 sequences, each of which contained 128 bits, in the present study. The p-values were considered as random when they exceeded 0.01. The randomness of the binary sequences generated from PUFs was verified by all of these tests. Collectively, these results indicate that the proposed PUF system is well-suited for cryptographic applications, secure authentication, and anticounterfeiting measures, as it exhibits a high degree of randomness.

The binarization results in the loss of valuable information in the interconnected structure of labyrinth-like features with rich entropy. The effective differentiation of these features is necessary for the direct use of these interconnected regions. The original images were subjected to the Canny edge detection algorithm for this purpose, resulting in the acquisition of a black contour of labyrinths in a white background for each image (Figure 5a,b). The edge data obtained were used in the calculation of Jaccard similarity. By computing the Jaccard similarities between image pairs, it was demonstrated that each sample possesses a unique structure and can be clearly distinguished from the others. As a result of applying this analysis to the entire image set, a Jaccard similarity matrix was obtained (Figure 5c), where a value of 1.0 is observed when a sample is compared with itself. In contrast, low similarity values between different samples confirm the system’s discriminative capability and the unclonable nature of the samples. This method was employed to quantitatively verify that the samples in the data set are distinct and unique from one another.

**Figure 5:**
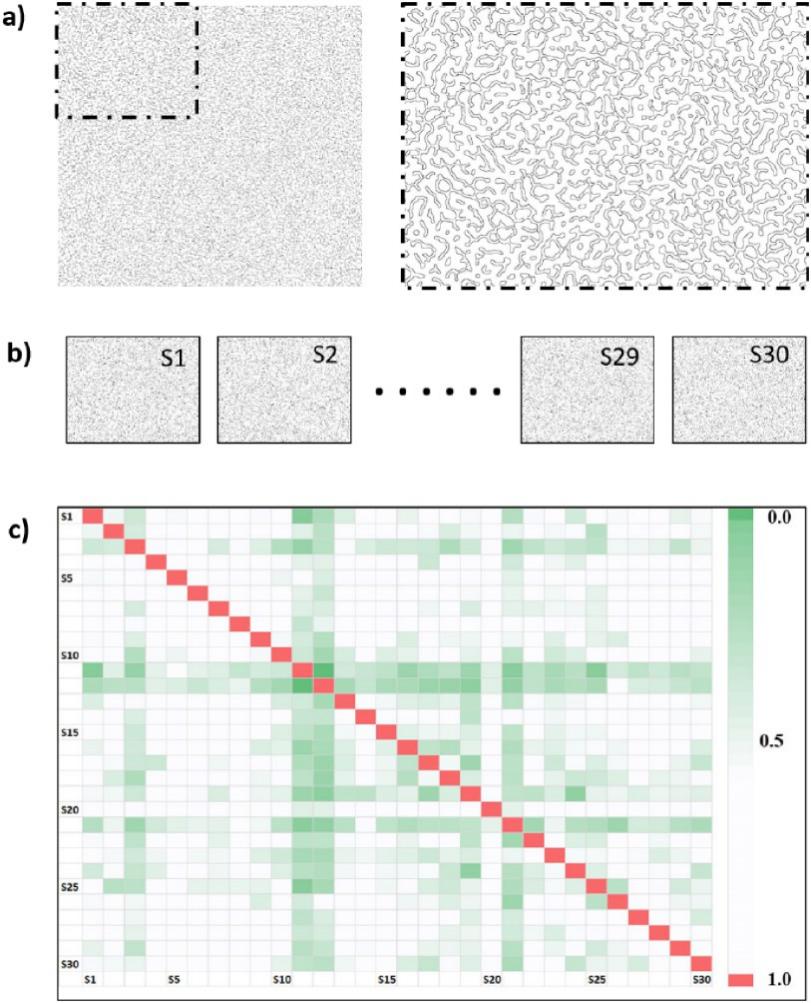
Comparison of Canny edge sets using Jaccard similarity. (a) Image obtained after Canny edge processing. (b) Canny edge processing for all samples. (c) Jaccard similarity matrix.

Feature matching algorithms and deep-learning methodologies enable direct and effective authentication of labyrinth-like features. First, the ORB algorithm was used for keypoint detection and matching, and similarity rates between query and reference images were calculated, demonstrating the system’s capability to distinguish between genuine and counterfeit tags. The ORB key point detection algorithm was utilized, which identified and stored keypoints in each image as objects. ORB provides scale-independent operation along with rotation, and zooming in and out.^59^ ORB employs these detector and descriptor algorithms for efficient feature matching, which have been shown capable of a range of applications, including PUFs.^42^ The image in the database was then matched to the query image (), and the matching points are depicted in as yellow lines. As an example, shows an image that is not in the database. One of the practical challenges encountered throughout the authentication process is the limitations related to imaging conditions, particularly those that arise depending on the spatial positioning of the image. Such issues are often addressed using physical markers that may be incorporated either before or after PUF fabrication. Similarly, factors such as illumination, brightness level, contrast, and the image rotation angle employed during imaging must be adequately defined to ensure reliable authentication. The similarity rates between the images of real and fake samples under different conditions were calculated (see the for details). The matching image pairings () and similarity rate distributions () of tags captured under various scenarios revealed a minimum similarity rate of 0.82. In contrast, fake tags had similarity rates of less than 0.002, validating the discriminating capacity of the system.

Subsequently, image classification and authentication were performed using a CNN model based on the ResNet-50 architecture, and the overall accuracy of the model was evaluated under various imaging conditions. The CNN, methodology based on the ResNet-50 architecture had been implemented in the MATLAB environment for recognition and authentication of labyrinthine patterns (Figure 6). The BoF technique was performed to extract features from the training data. The performance of the CNN-based model was evaluated after each training cycle by testing it on validation data under varying imaging conditions, and training accuracy graphs were generated (). Following the training phase, the authentication performance of PUFs was evaluated using CNN and image recognition algorithms. As illustrated in Figure 6, features extracted from genuine PUF images (Figure 6a, a1) were directly matched with features from user-captured images under different conditions (Figure 6b, b1–b5) and counterfeit images (Figure 6c, c1–c5), enabling robust identity authentication. The CNN-based model identified authentic samples with an average accuracy of 85.69% across a range of imaging settings (Figures 6d,e and ). The recognition of a single image from a database comprising 1000 images was completed in less than 300 ms using a standard laptop computer. The combined evaluation of both methods highlights the speed and lightweight advantages of traditional algorithms as well as the high accuracy and generalization capability of deep learning approaches, offering a versatile and reliable solution for PUF-based authentication systems.

**Figure 6:**
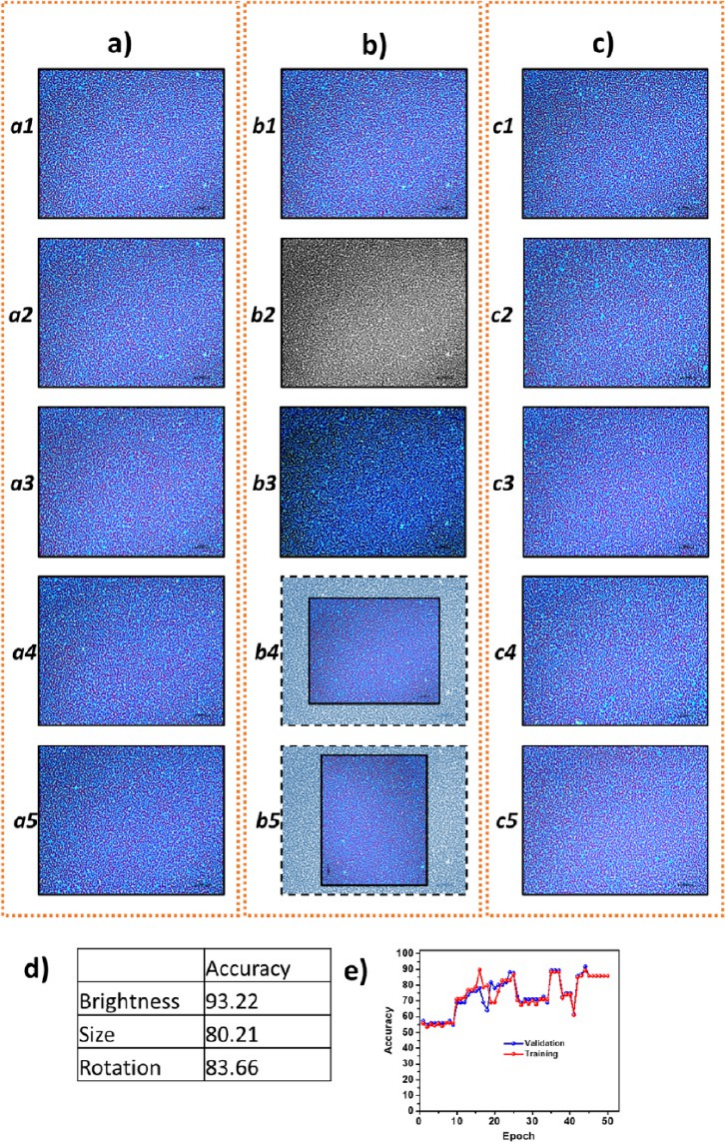
Authentication of PUFs with a CNN-based algorithm: (a) Images in the database. (b) Images taken by the user under different conditions. Images taken at different brightness (b2, b3), reduced size (b4), and rotation (b5). (c) Images not in the database (c1–c5). (d) Accuracy rates for identifying genuine samples using images taken under varied brightness, sizing, and rotation conditions. (e) Accuracy of the training and validation process of PUFs.

### 3.5 Entropy Analysis and Encoding Capacity

To quantitatively validate the entropy and encoding capacity imparted by the unique labyrinthine morphologies for PUF applications, a detailed analysis and calculations based on the statistical properties of the derived PUF keys was conducted (see the for details). This methodology is a well-established and validated approach commonly used in the performance evaluation of similar PUF systems.^2,7^ The quantitative Shannon entropy analysis reveals that our keys possess a high degree of randomness and unpredictability. The average Shannon entropy (*H­(X))* of the keys was calculated to be 0.997 bits/bit, a value remarkably close to the ideal of 1,^2,43,60,61^ while the average min-entropy *(H*_*min*_ *(X))* was calculated to be 0.931 bits/bit. This finding indicates that the information content of the keys is maximized, rendering them practically impossible to predict with a deterministic model. Furthermore, a low mutual information (*MI*) value of 0.0274 bits/bit between bits confirms a high degree of statistical independence. This characteristic provides robust protection against potential side-channel attacks that rely on interbit dependencies. Finally, the calculated average value of 2.09 bits for the Hausdorff distance *(d*_*H*_ *(A,B))* between keys empirically proves that each generated key is distinct and unique.^2^ The encoding capacity and storage density were also quantitatively evaluated. The encoding capacity (*N*_*keys*_) of the PUF keys reaches ∼2^216^, a figure indicating that the potential number of unique keys is sufficiently large. Additionally, the storage density (*D*_*storage*_) which represents the amount of unique information per unit area, was determined to be ∼19692 bits/mm^2^. This data supports that the PUF structure utilizes its physical area with high efficiency, presenting an ideal solution for compact security tags.^2,7,60^ The current labyrinthine pattern offers robust authentication due to its highly favorable entropy and encoding capacity. Compared to recently reported PUFs with interconnected patterns, it stands out for its fabrication simplicity, requiring only spin-coating and thermal annealing under ambient conditions (see Table S3, Supporting Information for a comparison with recent PUFs).^2,4,39,42–47^

### 3.6 Stability of PUFs

Stability is essential for real-world applications of *molecular*-PUFs. An ideal *molecular*-PUF should endure environmental effects and maintain consistent performance over an extended time frame. To evaluate the stability characteristics of our newly developed *molecular*-PUFs, we subjected them to a range of stress conditions. Initially, the maximum temperature at which the labyrinth-like features retained structural integrity was determined. As shown in , the PUF features, derived from our molecularly engineered compound optimized for high-temperature resilience, maintained excellent structural integrity up to 150 °C. Closer analysis indicated that this temperature marks the onset of thermally induced dewetting in localized regions, with complete structural disintegration occurring at 180 °C. Next, the *molecular*-PUF labels were subjected to thermal stability, humidity, water immersion, and UV-light exposure tests to assess their performance under realistic environmental stresses. For each test, optical images of the same region, identified using a physical marker, were compared before and after exposure. Performance degradation was quantitatively evaluated by comparing conventional binary keys generated from the images and by directly analyzing image differences using a feature-matching algorithm. The thermal stability test, conducted at 60 °C for 48 h, and the humidity stability test, performed at 80% relative humidity (50 °C) with constant vapor exposure for 48 h, showed impressive overall similarities of 91.7% and 96.79% (based on the feature matching algorithm), respectively (). The water immersion test was performed by submerging the samples in water for 48 h, which yields a high overall similarity of 94.42% based on the feature matching algorithm (). Finally, after 48 h of continuous UV-light exposure (10 W, 365 nm wavelength), the *molecular*-PUF samples exhibited an overall similarity of ∼86.08% based on the feature matching algorithm (). The high similarity values observed in these stability tests suggest the significant potential of **InIm-BF**_**2**_ labyrinthine *molecular*-PUFs for real-world applications.

## 4 Conclusions

A high-dipole moment (4.72 D) small molecule, **InIm-BF**_**2,**_ was developed as a novel *molecular*-PUF material. A simple two-step process, spin-coating followed by thermal annealing at 90 °C under ambient conditions, produced nanoscopic molecular thin films with intricate labyrinthine patterns exhibiting excellent PUF characteristics confirmed via multiple image analysis and computational algorithms. **InIm-BF**_**2,**_ a difluoroborate complex of an indolyl-imine ligand, has a dipolar, coplanar π-backbone exhibiting antisymmetric cofacial arrangement with π–π stackings of 3.86 Å. These properties, combined with short C–H···π contacts (2.74–2.88 Å) and nonclassical C–H···F hydrogen bonds (2.47–2.51 Å, 23.4% of the Hirshfeld surface), drive the formation of interconnected, irregularly shaped patterns. The molecule’s highly polar nature prevents complete dewetting and promotes the formation of micron-sized (≈50–100 μm) nearly amorphous thin film features having disordered, short-range intermolecular interactions, primarily π–π stackings. Unlike randomly positioned isolated features used in classical binarized keys, the interconnected labyrinthine structures exhibit rich entropy and complex features which can be directly authenticated via deep-learning methodologies. Our results indicate that the proposed boron-based PUF system is well-suited for cryptographic applications, secure authentication, and anticounterfeiting measures. From a *molecular*-PUF design perspective, boron-based molecular family offers a significant potential for designing new molecules with tailored dipole moments, intermolecular interactions, and thin film self-assembly properties for advanced PUF applications. Furthermore, designing molecular systems with tailored temperature responsiveness and programmed heating/cooling cycles could be a promising future direction for generating reconfigurable *molecular*-PUFs.^62^ The unique photophysical properties, including fluorescence profiles and excited-state decay dynamics, and structural characteristics, such as Raman mapping, offer potential for multiplex encoding to enhance security. This study demonstrates a promising, scalable approach to fabricating high-entropy PUF patterns and provides key insights into designing novel organic materials for advanced security applications.

## Supplementary Material





## Notes

Molecular strings (CIF)
^1^H/^13^C NMR and MALDI TOF-MS spectra (Figures S1–S6); DSC plots (Figure S7); photophysical properties (Figure S8); single crystal X-ray data (Table S1); PUFs on different substrates (Figure S9); large-area fabrication (Figure S10); PUF authentication model (Figures S11, S12); optical microscope images and binary keys (Figure S13); PUF performance analysis and parameters (Figure S14 and Table S2); performance metrics for interconnected PUFs (Table S3); authentication, similarity data, and accuracy (Figures S15–S17); PUF stability (Figures S18–S22) (PDF)
