# Supplementary material for: Labyrinthine Microstructures with a High Dipole Moment Boron Complex for Molecular Physically Unclonable Functions
Source: ACS Appl Mater Interfaces. 2025 Oct 29;17(45):62685–96. doi: 10.1021/acsami.5c13228 (PMC12616588; doi:10.1021/acsami.5c13228)
Supplement: Supplementary file 2 [file am5c13228_si_002.pdf]

Supporting Information

Labyrinthine Microstructures with a High Dipole-Moment Boron Complex for Molecular Physically Unclonable Functions

Tevhide Ayça Yıldız[1], *N.Burak Kiremitler*[2,3], *Nilgun Kayaci*[1], *Mustafa Kalay*[2,4], *Emrah Özcan*[5,6], *İbrahim Deneme*[1], *Zehra Coşkun*[5], *Mustafa Serdar Onses*[2,3*], *Bünyemin Çoşut*[5*], *Hakan Usta*[1*]

[1] Department of Materials Science and Nanotechnology Engineering, Abdullah Gül University, 38080 Kayseri, Türkiye.

[2] ERNAM - Nanotechnology Research and Application Center, Erciyes University, Kayseri, 38039, Türkiye.

[3] Department of Materials Science and Engineering, Erciyes University, Kayseri, 38039, Türkiye.

[4] Department of Electricity and Energy, Kayseri University, Kayseri, 38039, Türkiye.

[5] Department of Chemistry, Faculty of Science, Gebze Technical University, Gebze, Kocaeli 41400, Türkiye.

[6] Department of Physics, Faculty of Science, University of South Bohemia, Branišovská 1760, České Budějovice 370 05, Czech Republic.

*Address correspondence to: hakan.usta@agu.edu.tr (HU), bc@gtu.edu.tr (BC), onses@erciyes.edu.tr (MSO).

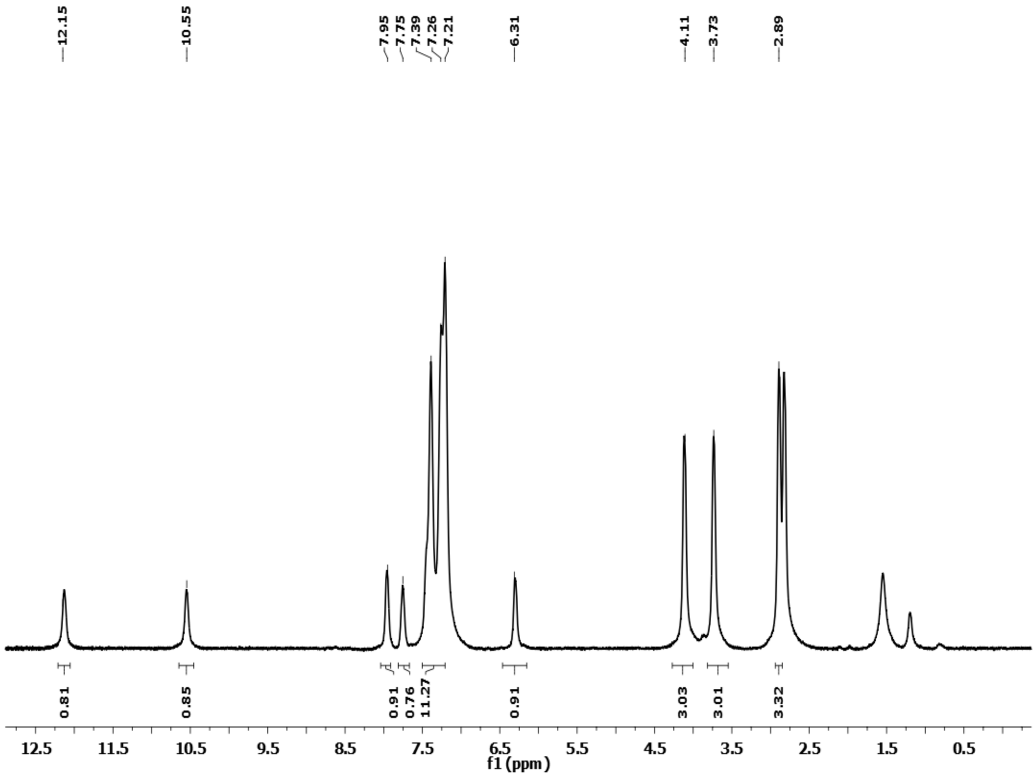

Figure S1. ^{1}H-NMR spectrum of **InIm** in $CDCl_3$.

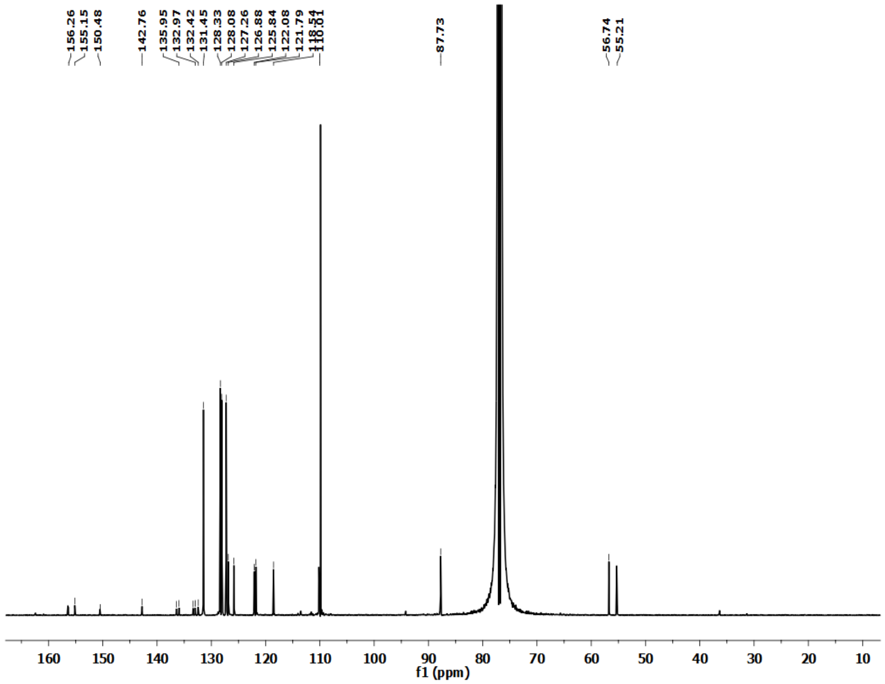

Figure S2. ^{13}C-NMR spectrum of **InIm** in $CDCl_3$.

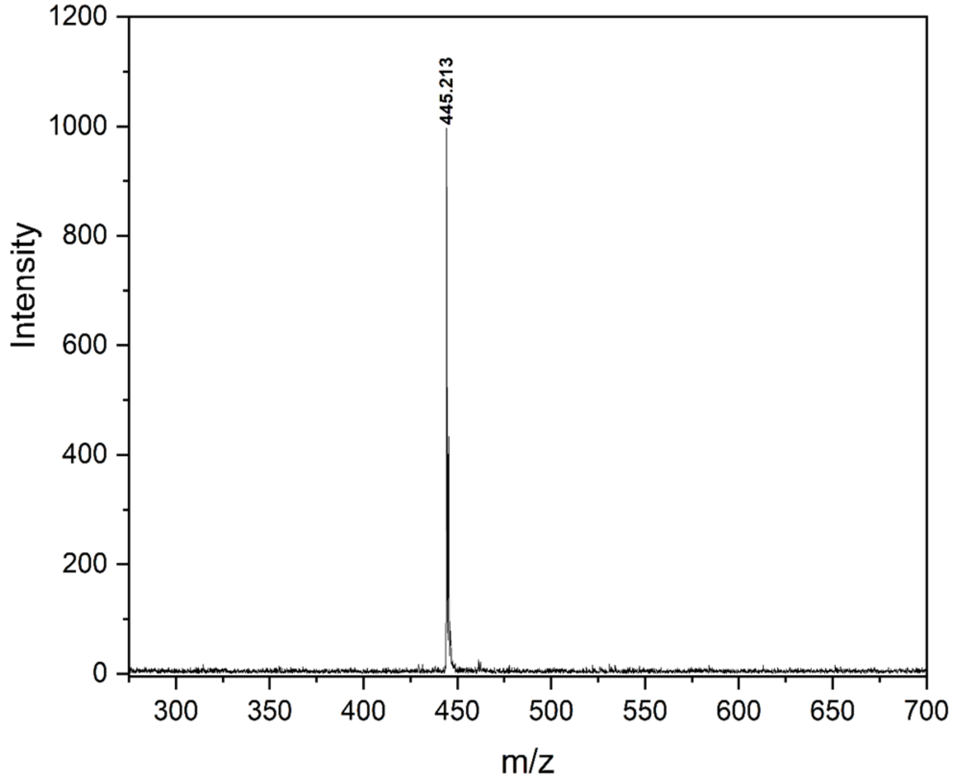

Figure S3. Positive ion and linear mode MALDI TOF-MS spectrum of **InIm**.

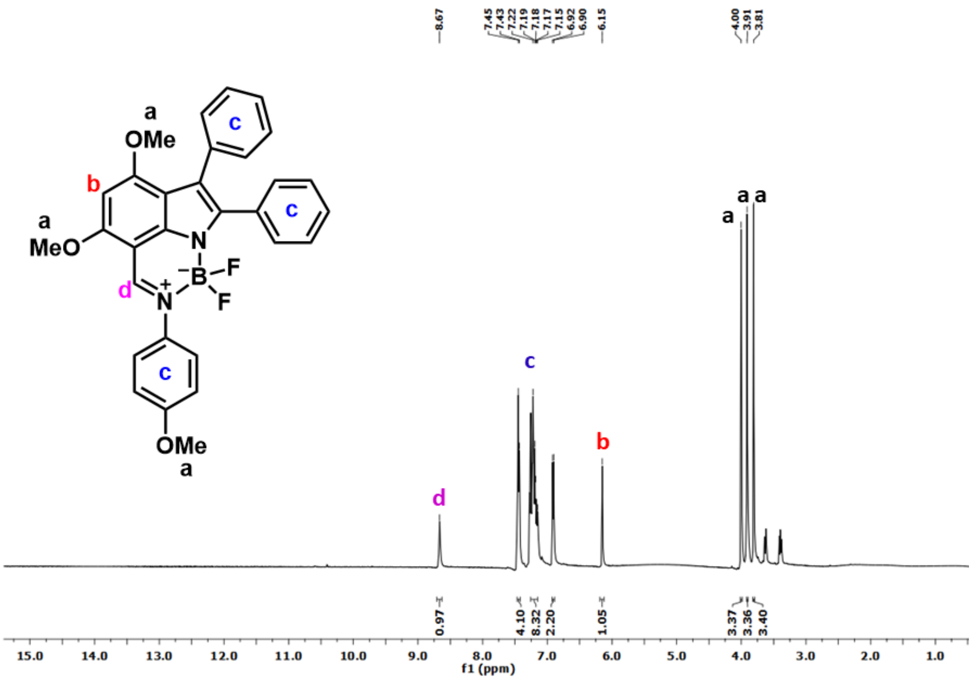

Figure S4. ^{1}H-NMR spectrum of **InIm-BF₂** in $CDCl_3$.

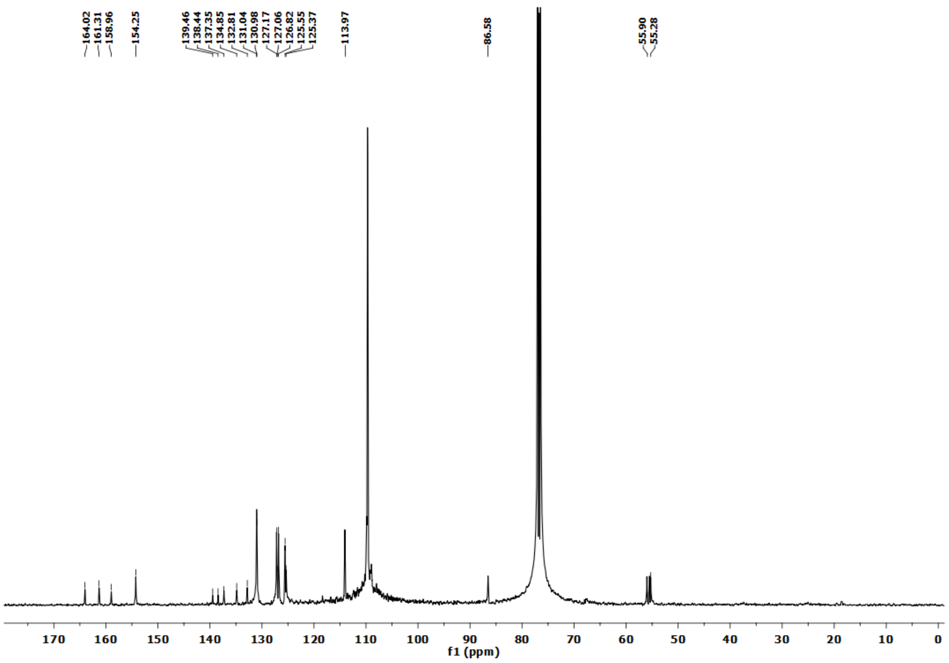

Figure S5. ^{13}C-NMR spectrum of **InIm-BF$_2$** in $CDCl_3$.

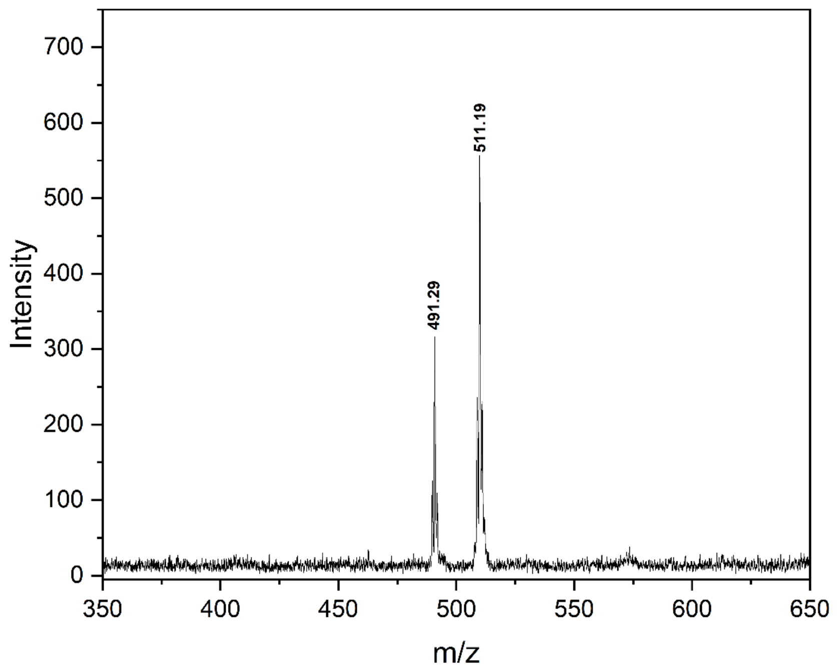

Figure S6. Positive ion and linear mode MALDI TOF-MS spectrum of **InIm-BF$_2$**.

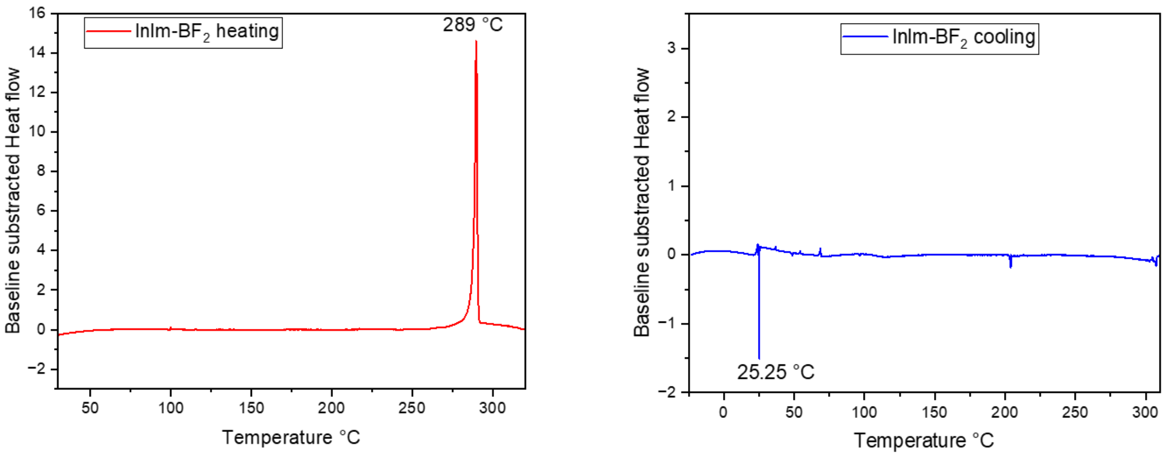

Figure S7. Heating (red) and cooling (blue) curves of **InIm-BF₂** measured under nitrogen (temperature ramp = 10 °C/min) with differential scanning calorimetry (DSC).

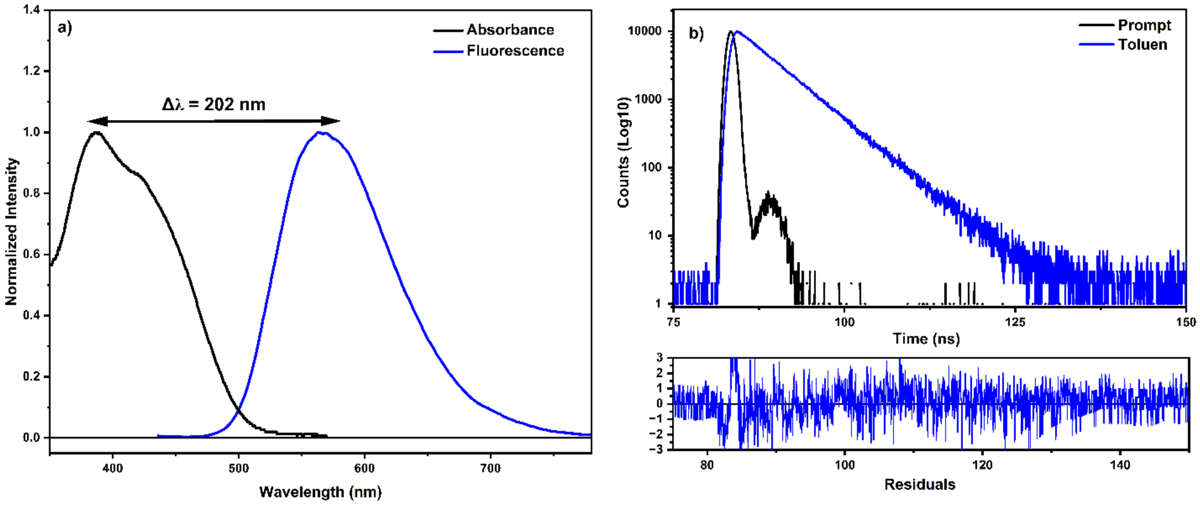

Figure S8. (a) Normalized absorption (left) and fluorescence (right) spectra of **InIm-BF₂** in toluene (1×10^{-5} M; excitation: 420 nm). (b) Fluorescence decay profile of **InIm-BF₂** in toluene upon excitation at 390 nm.

X-ray data collection and structure refinement

Unit cell measurements and intensity data collection was performed on an Bruker APEX II QUAZAR three-circle diffractometer using monochromatized Mo *Kα* X-radiation (λ = 0.71073 Å). Indexing was performed using APEX2 [1]. Data integration and reduction were carried out with SAINT V8.34A [2]. Absorption correction was performed by multi-scan method implemented in SADABS V2014/5 [3]. The structures were solved and refined using the Bruker SHELXTL Software Package [4]. All non-hydrogen atoms were refined anisotropically using all reflections with $I > 2\sigma(I)$. The C-bound H atoms were positioned geometrically and refined using a riding mode. The N-bound H atoms were located from the difference Fourier map and restrained to be 0.89 Å from N atom using DFIX and their position were constrained to refine on their parent N atoms with Uiso(H) = 1.2Ueq(N). Furthermore, the unit cells of compound **InIm-BF$_2$** had a disordered solvent molecule which couldn't be modelled. Therefore, the SQUEEZE command of PLATON [5] was used for removing solvent molecules and then the rest of the molecules were refined without the solvent. The final geometrical calculations and the molecular drawings were carried out with Platon (version 1.17) and Mercury CSD (version 3.5.1) program [5,6]. CIF was deposited with the Cambridge Crystallographic Data Centre (CCDC 2469750 for InIm-BF$_2$)

Table S1. Crystal data and refinement parameters for compound **InIm-BF_2.**

**Empirical Formula**	$C_{30}H_{25}BF_2N_2O_3$
**Formula weight/g. mol^{-1}**	510.33
**Temperature/K**	173.01
**Wavelength (Å)**	MoKα (λ = 0.71073)
**Crystal System**	Monoclinic
**Space group**	Pc
**a/Å**	12.841(5)
**b/Å**	27.674(10)
**c/Å**	7.714(3)
**α/°**	90
**β/°**	105.021(5)
**γ/°**	90
**Crystal size/mm^3**	0.321 × 0.217 × 0.108
**V/ Å^3**	2647.7(16)
**Z**	2
**ρcalcd (g. cm^{-3})**	1.280
**μ (mm^{-1})**	0.092
**F(000)**	1064.0
**θ range for data collection/°**	1.472 to 50.05
**h/k/l**	$-15 \leq h \leq 15$, $-32 \leq k \leq 32$, $-9 \leq l \leq 9$
**Reflections collected**	28521
**Independent reflections**	9145 [R_{int} = 0.0564
**Data/restraints/parameters**	9145/2/691
**Goodness-of-fit on F^2 (S)**	1.326
**Final R indices [I > 2σ(I)]**	R_1 = 0.0973, wR_2 = 0.2794
**R indices (all data)**	R_1 = 0.1019, wR_2 = 0.2871
**Largest diff. peak/hole / e Å^{-3}**	0.85/-0.51

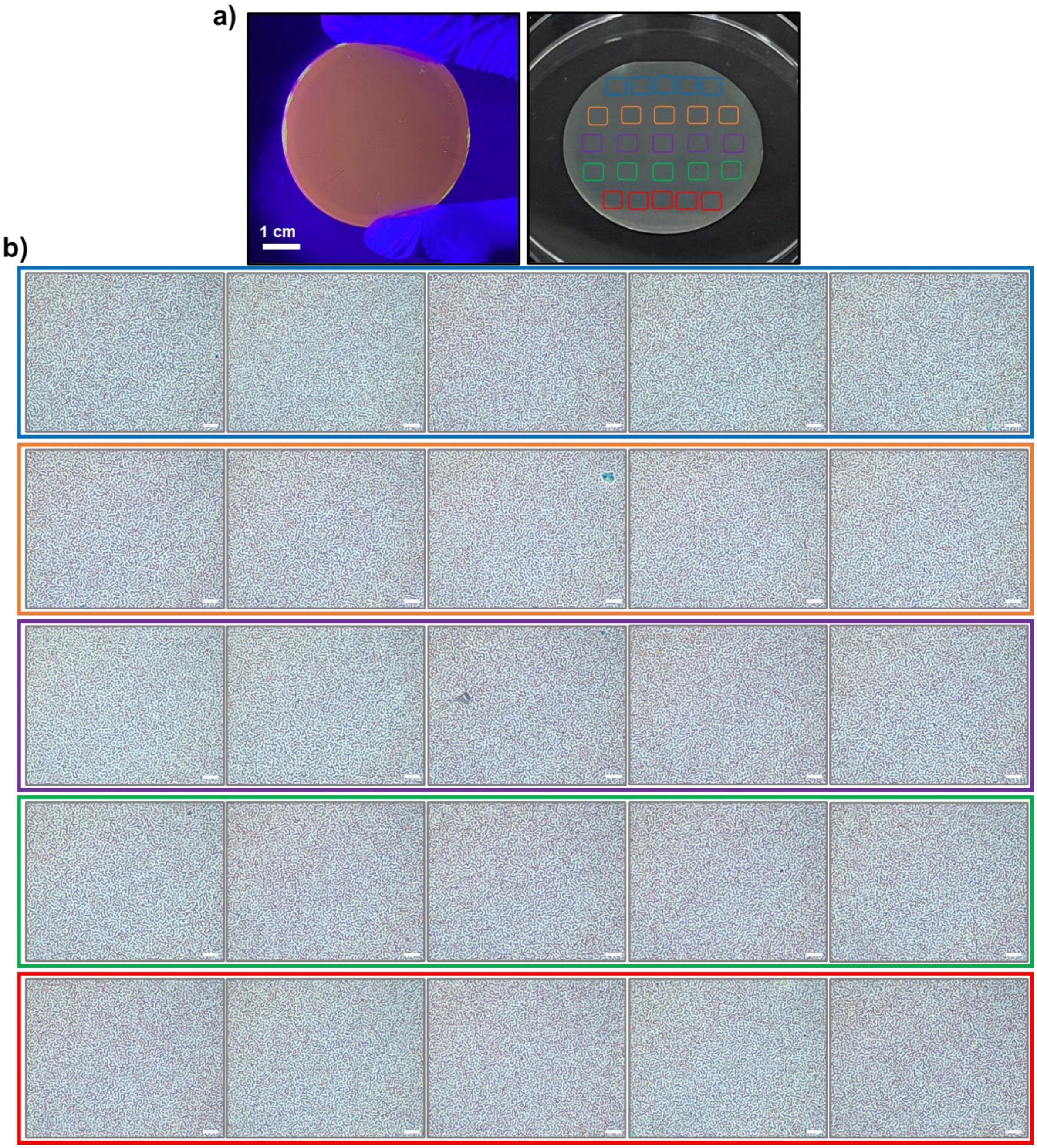

Figure S9. Demonstration of large-scale fabrication of *molecular*-PUFs. (a) Photographs of a 2-inch silicon wafer patterned with labyrinthine formations, shown under UV (left) and visible (right) illumination, with a schematic indicating the 25 distinct regions selected for microscopic analysis. (b) Representative optical microscope images captured from the 25 specified regions on the wafer, confirming the uniformity of the labyrinth structures across the entire surface. Scale bars: 10 μm.

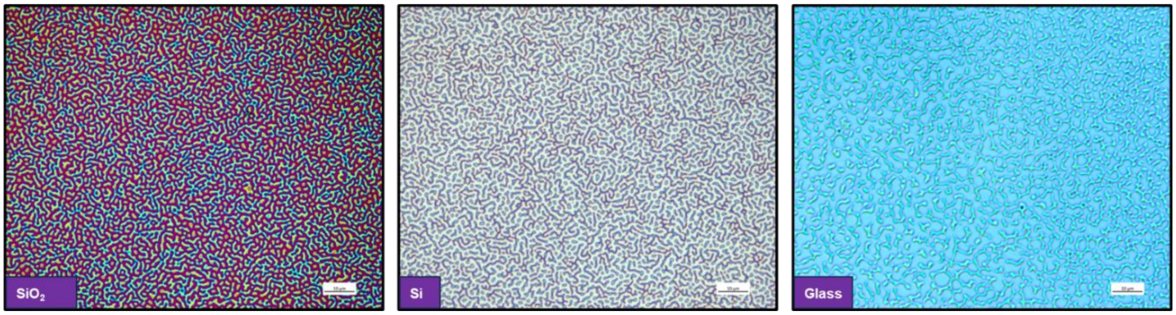

Figure S10. Optical microscope images of *molecular*-PUFs fabricated on (from left to right) silicon substrate with a 300 nm oxide layer, silicon substrate with only a native oxide layer, and microscope glass.

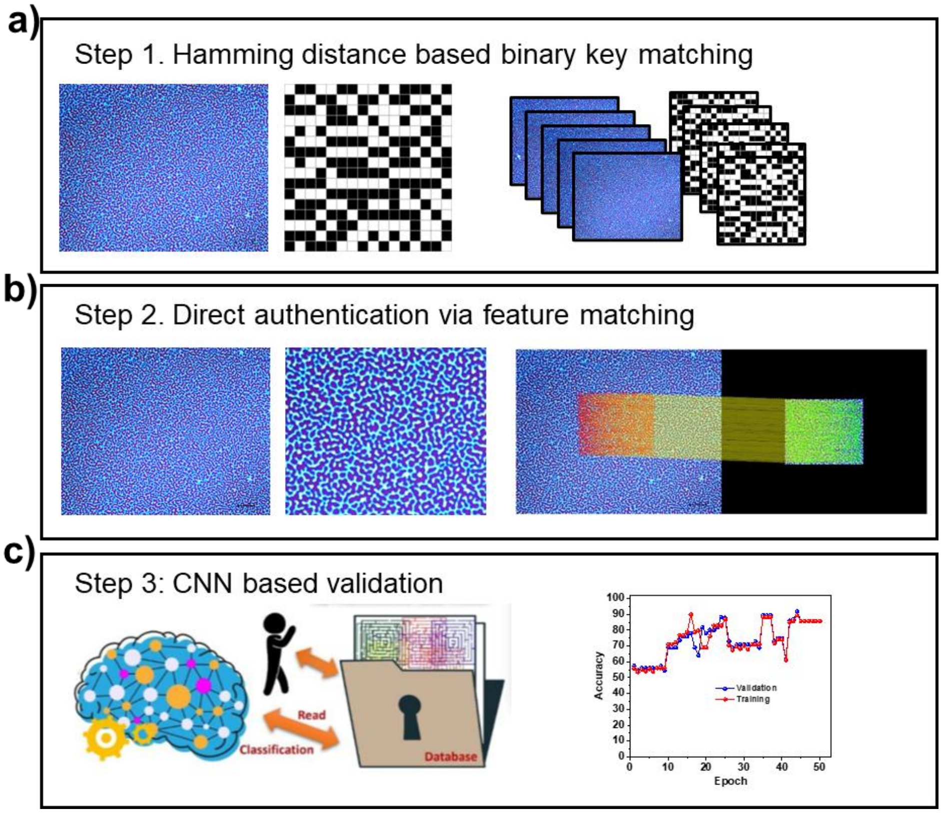

Figure S11. Schematic of the multi-layered authentication approach. (a) PUF key generation and binary matching based on Hamming Distance. (b) Feature matching stage utilizing the ORB algorithm. (c) Final verification of pattern integrity using a CNN model.

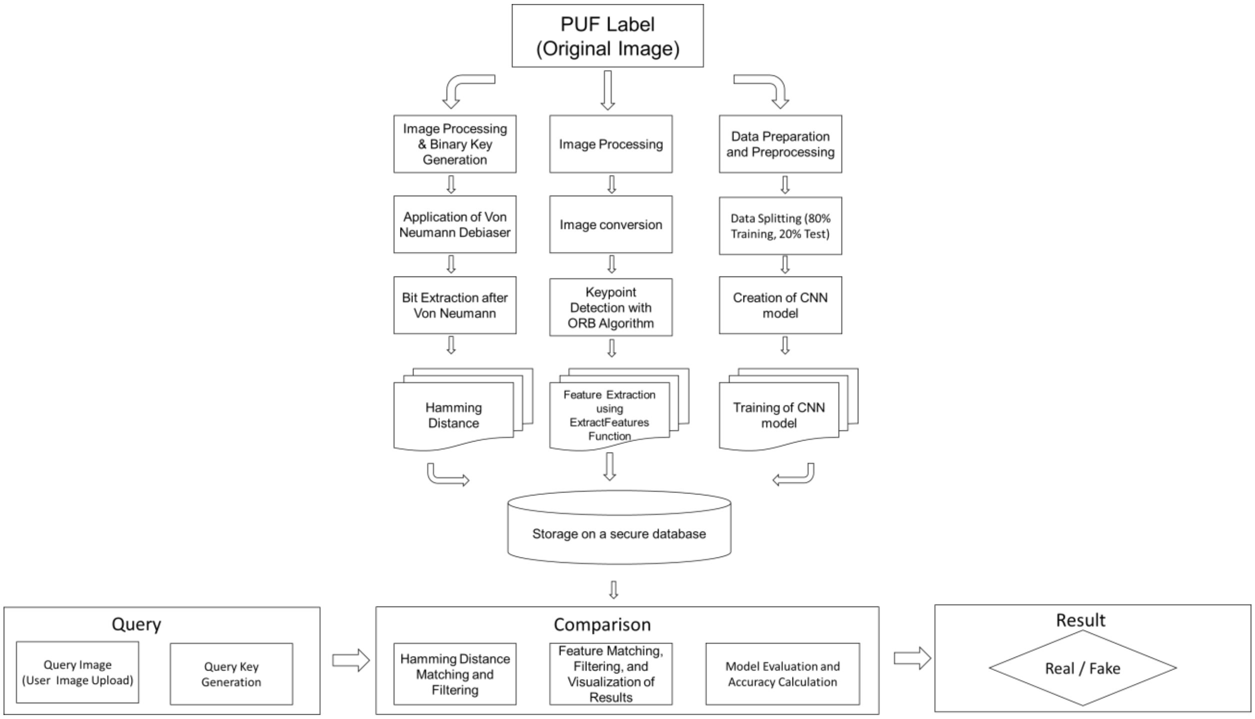

Figure S12. A multi-modal workflow for PUF authentication, illustrating the enrollment and query phases.

PUF Performance Analysis

Uniformity

The uniformity metric is calculated using the following equation (1).

Uniformity = $\frac{1}{n}\sum_{i=1}^{n} r_{i,l}$ x100% (1)

n**:** The number of bits

r_l**:** Ith binary bit (0 or 1) of an *n-bit* response from a key.

Uniqueness Equation (2):

Uniqueness = $\frac{2}{s(s-1)}\sum_{i=1}^{s-1}\sum_{j=i+1}^{s}\frac{HD(R_i,R_j)}{n}$ (2)

S: The number of keys from different chips.

HD (R_i, R_j) : Hamming distance between chips *i* and *j*.

Normalized Hamming distance = Hamming distance divided by the number of bits, $\frac{HD(R_i,R_j)}{n}$

Reliability Equation (3):

HD_{INTRA} = $\frac{1}{m}\sum_{t=1}^{m}\frac{HD(R_i,R'_{i,t})}{n}$ (3)

m: number of samples studied under different conditions.

Reliability = 1.00-HD_{INTRA}

Classic von Neumann Debiasing:

The following procedure was used for debiasing.

• Consider the key is composed of consecutive pairs of bits

• Discard the pair of bits in the case of bits consisting of 11 or 00

• Retain the first bit, in the case of bits consisting of 10 or 01

Similarity Rate Calculation

$$\frac{N_m}{N_t}$$

N_m represents the number of matching features and N_t represents the total number of features.

Entropy Analysis and Encoding Capacity

Shannon Entropy

Shannon Entropy (*H(X)),* is a fundamental concept that measures the uncertainty or information content of a random variable. In a binary system composed of 0s and 1s, the entropy of a bitstring was calculated using the following equation (4):

$$H(X) = -\big(p \cdot log_2(p) + (1-p) \cdot log_2(1-p)\big) \ (4)$$

p represents the probability of '1's occurring in the string. For an ideally random string, *p* =0.5, which allows the system to achieve the maximum entropy of *H(X)* =1 bits/bit.

Min-Entropy

Min-entropy, or $(H_{min}(X))$, quantifies the randomness of an information source under a worst-case scenario. It is based on the probability of the most likely outcome and is utilized to establish a lower bound for security. For a binary string, min-entropy was defined using the following equation (5):

$$H_{min}(X) = -log_2(max(p, 1-p)) \ (5)$$

p again signifies the probability of '1's.

Mutual Information

Mutual Information (MI) is a measure of the statistical dependence between two random variables. In the context of PUF keys, this metric is employed to determine whether two distinct bit positions are interdependent. The mutual information, $MI(X, Y)$, between two bits, was calculated with the following equation (6):

$$MI(X,Y) = \sum_{x\in\{0,1\}} \sum_{y\in\{0,1\}} p(x,y). log_2 \left(\frac{p(x,y)}{p(x).p(y)}\right) \ (6)$$

$p(x, y)$ is the joint probability of X and Y, while $p(x)$ and $p(y)$are their respective marginal probabilities. Ideally, the MI value should be close to 0, which indicates that the bits are independent of one another.

Hausdorff Distance

The Hausdorff distance $(d_H(A,B))$, is a metric that measures the greatest distance between two geometric sets. For PUF keys, it is used to assess the similarity or dissimilarity between the sets of bit positions (specifically, the positions of '1's) of two keys. The Hausdorff distance between two binary keys, *A* and *B*, was calculated by the equation (7):

$$d_H(A,B) = max\left\{\sup_{a\in A} \inf_{b\in B} d(a,b), \sup_{b\in B} \inf_{a\in A} d(b,a)\right\} \ (7)$$

A and *B* are the sets representing the positions of the '1' bits, and *d(a,b)* is the distance between two positions, typically $|a-b|$.

Encoding Capacity

Encoding capacity (N_{keys}), provides an estimate of the number of unique keys that a PUF can generate and it is calculated using the equation (8). It indicates the theoretical security limit of a PUF system:

$$N_{keys} \approx k^{m.L.(1-\beta)} \qquad (8)$$

k: The number of bits (for a binary system, *k=2*)

m: The number of key types (if only one type of PUF key is used, *m=1*)

L: The key length in bits

β: The effectiveness threshold

Storage Density

The storage density ($D_{storage}$), refers to the number of unique bits stored per unit of area and it is calculated using the equation (9). This metric indicates the physical efficiency of the PUF in terms of its size

$$D_{storage} = \frac{L}{A_{area}} \qquad (9)$$

L: The key length in bits

A_{area}: The area where the PUF key is generated

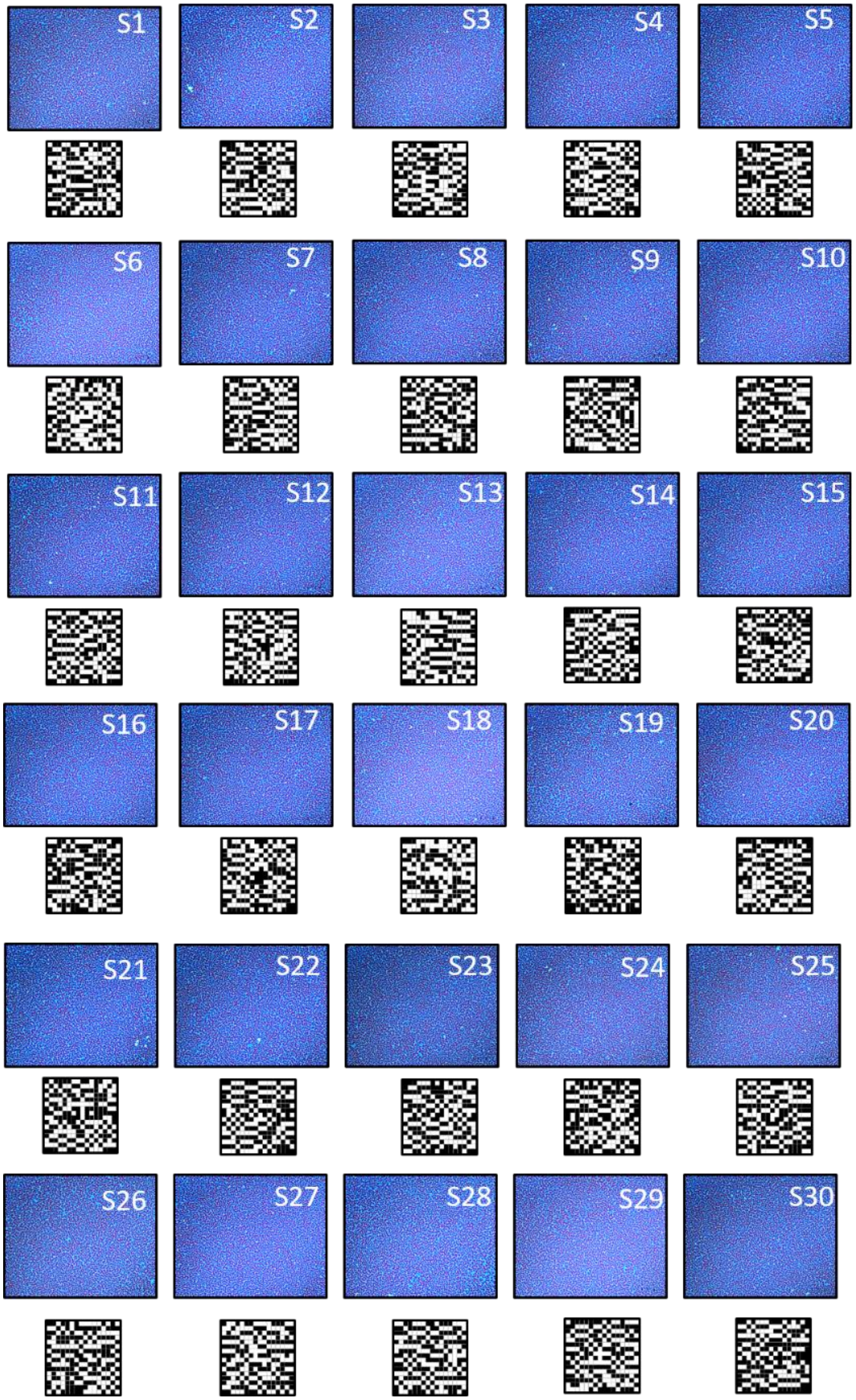

Figure S13. Optical microscope images of 30 different PUFs and their corresponding binary keys.

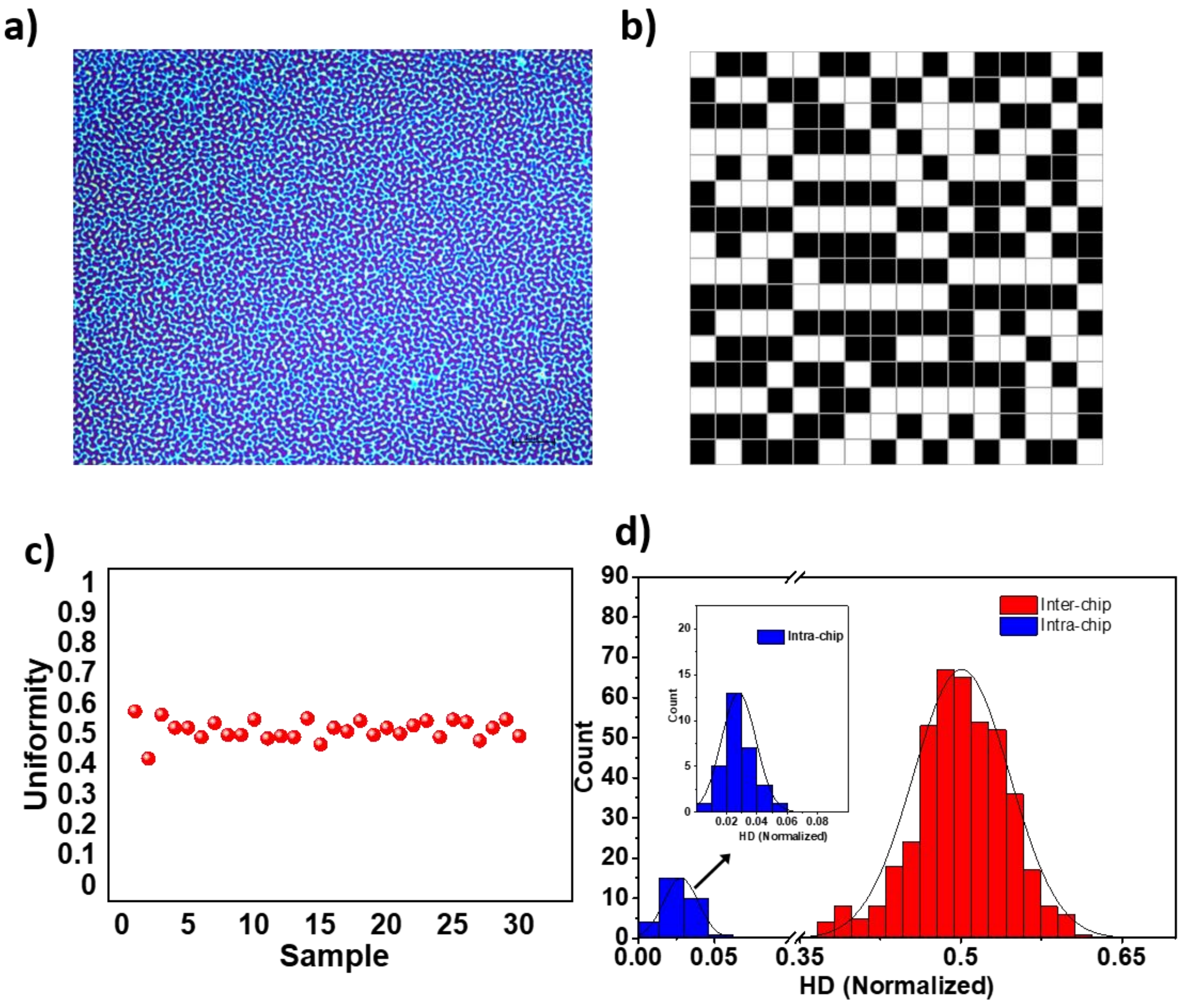

Figure S14. Evaluation of PUF performance. (a) Optical image of the sample used in PUF analysis. (b) Binary image corresponding to the optical image. (c) Uniformity of bits obtained from 30 samples. (d) distribution of HD_{INTER} and HD_{INTRA}.

Table S2. Randomness tests, p-values and pass rate values.

NIST Statistical Test	p-value		Proportion	Result
Frequency	0.5173		60/60	Pass
Block Frequency	0.013328		60/60	Pass
Cumulative sums	0.1344	0.15176	60/60	Pass
Runs	0.75124		59/60	Pass
Longest run of ones	0.26627		60/60	Pass
Approximate entropy	0.58262		60/60	Pass
Serial	0.44312	0.44648	60/60	Pass

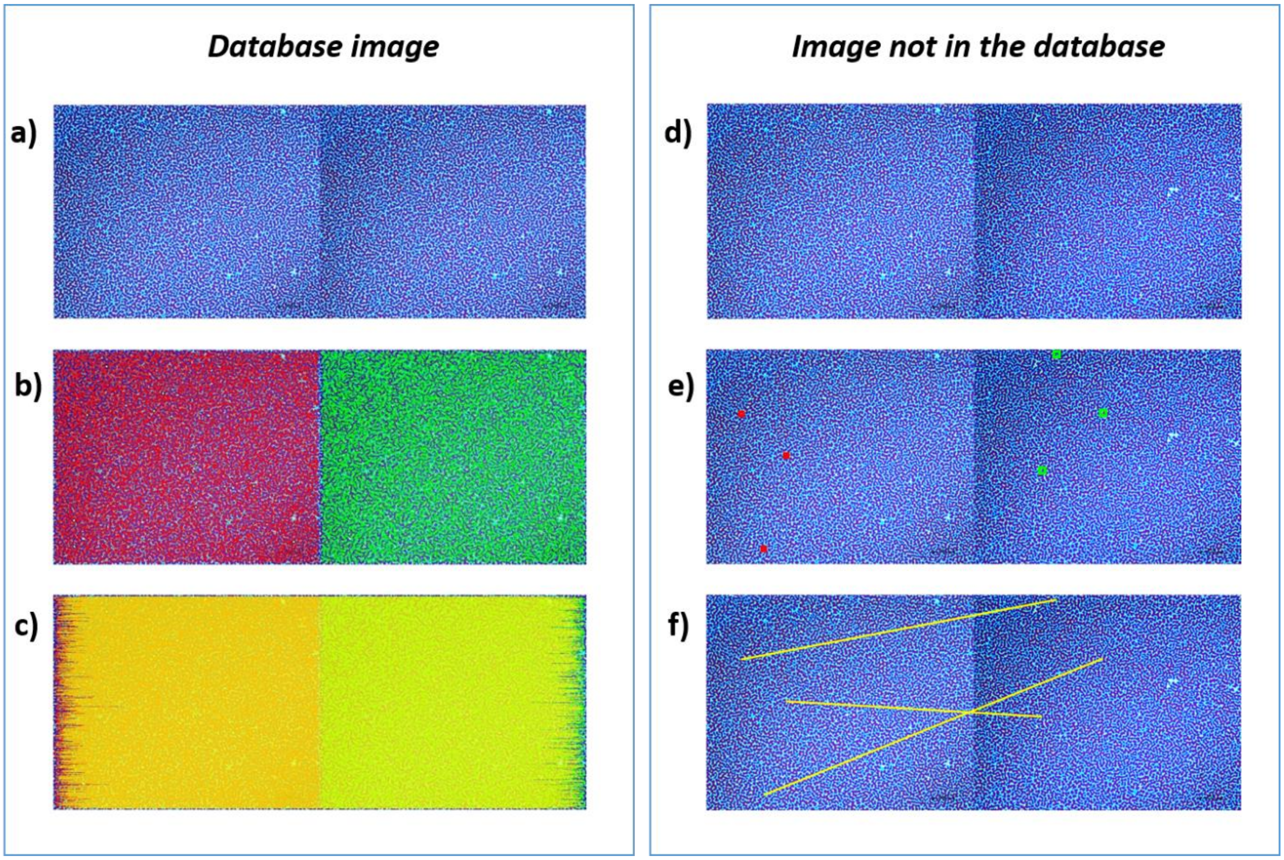

Figure S15. Authentication through the feature matching algorithm. (a) Matching images that are in the database (b) Identifying key points of matching images (c) Feature matching (d) Matching images that are not in the database (e) Identifying key points of matching images (f) Feature matching (In both cases, the yellow lines represent the feature match between the database and the user image).

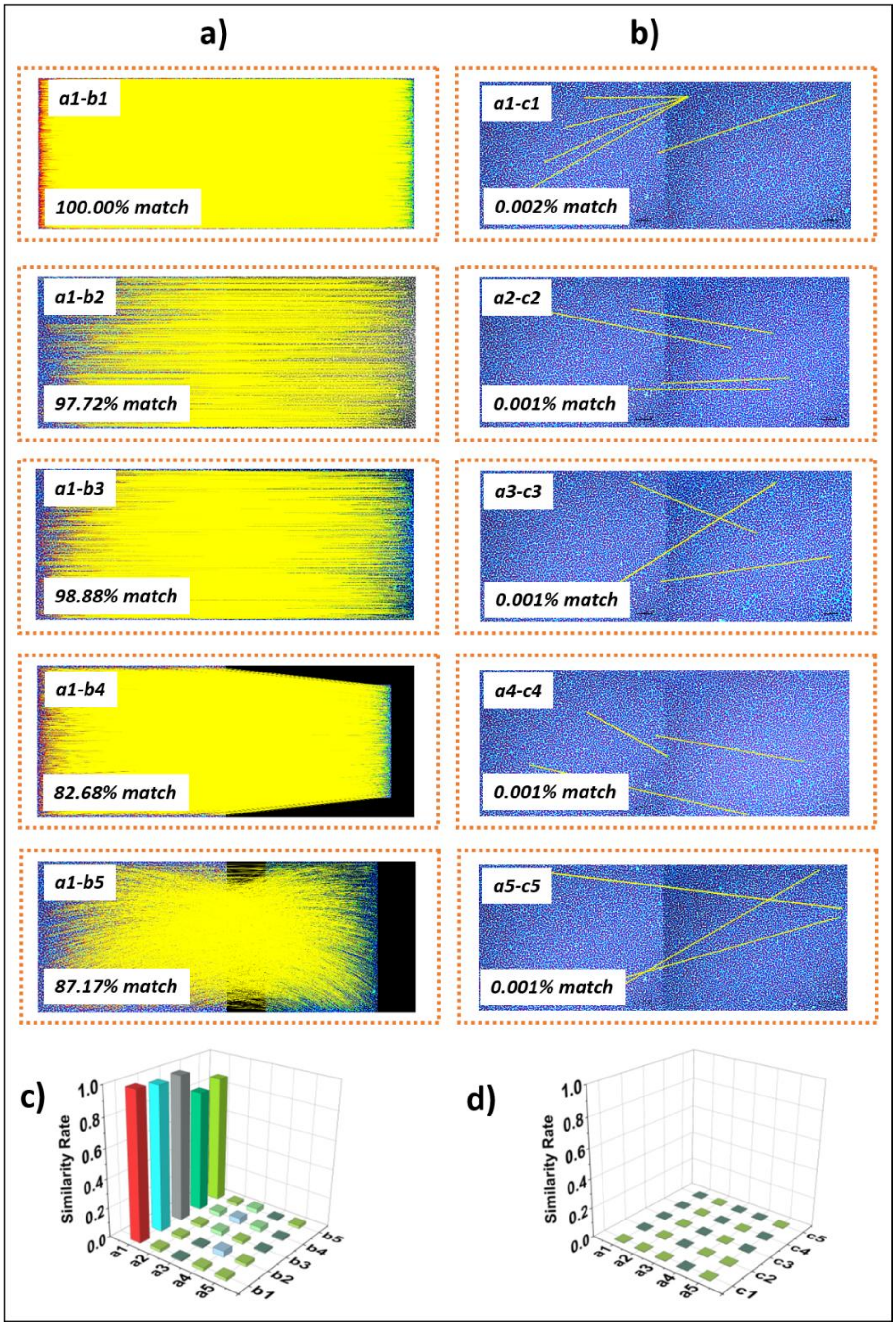

Figure S16. Similarity rates calculated between genuine images (a) Matching images in the database with images taken by the user (b) Matching of images in the database with (fake) images not in the database (c) Similarity ratio of real images (d) Similarity ratio of fake images.

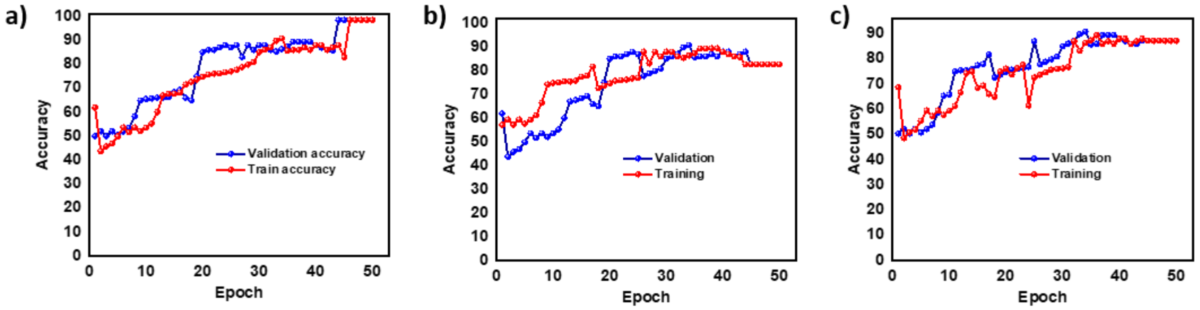

Figure S17. Accuracy for identifying genuine samples using images taken under varied (a) brightness, (b) size, and (c) rotation conditions.

Table S3. Characteristics and performance comparison of recently reported PUFs having interconnected patterns.

Material/Method	Read out mechanism	Molecular PUF	Performance Metrics	Reference
Polymeric microparticles with silica-film wrinkles (core-shell structure wrinkles)	Optical	No	Encoding capacity = 10^{135}	*Adv. Mater. 2015* [7]
Atomic layer infiltration of Al_2O_3 on screen printed PDMS film (PDMS/Al_2O_3 hybrid layer wrinkles)	Optical	No	Encoding capacity = 440^{64}	*ACS Appl. Mater. Interfaces, 2021* [8]
Birefringent wrinkled structure of liquid crystals	Optical (with two polarizers)	Yes	Shannon Entropy = 0.9793 ± 0.03 (row axis) and 0.9806 ± 0.02 (column axis)	*Light: Science & Applications, 2023*[9]
Supramolecular network of P4VP-nBA-S and DSP-OH (copolymer and small molecule)	Optical/ Fluorescence	Yes (Polymer-Molecule Hybrid)	Not reported	*Nat. Commun. 2020*[10]
Fractal-guided and thermally annealed Au film networks	Optical	No	Encoding capacity = 10^{348}	*Nat. Commun. 2023*[11]
Metallic nanopatterns replicated from self-assembled block copolymer nanotemplates	Electrical resistance, optical dichroism or Raman signals	No	Encoding Capacity = 2.83×10^{163}<br>Sample Entropy≈1.085<br>LZ Entropy≈0.3579<br>Permutation entropy≈0.93	*Nat. Electron.* 2022[12]
Spontaneous folding of ultrathin responsive gels integrated with plasmonic nanostructures	Optical/Raman	No	Not reported	*ACS Appl. Mater. Interfaces, 2016*[13]
Perovskite Quantum Dots Embedded in Spontaneous-Phase-Separated Polymers	Optical/ Fluorescence	No	Encoding Capacity = 10^{653}	*ACS Appl. Mater. Interfaces, 2025*[14]
Self-organization of anthracene-functionalized poly(styrene-block-butadiene-block-styrene)	Optical (Confocal laser microscopy) AFM	No	Encoding Capacity = 2^{16348}	*Adv. Funct. Mater. 2024*[15]
Labyrinthine Microstructures with a High Dipole-Moment Boron Complex	Optical	Yes	Encoding Capacity = 2^{216}<br>Shannon Entropy = 0.997<br>Min-Entropy = 0.931<br>Mutual Information = 0.0274	*This work*

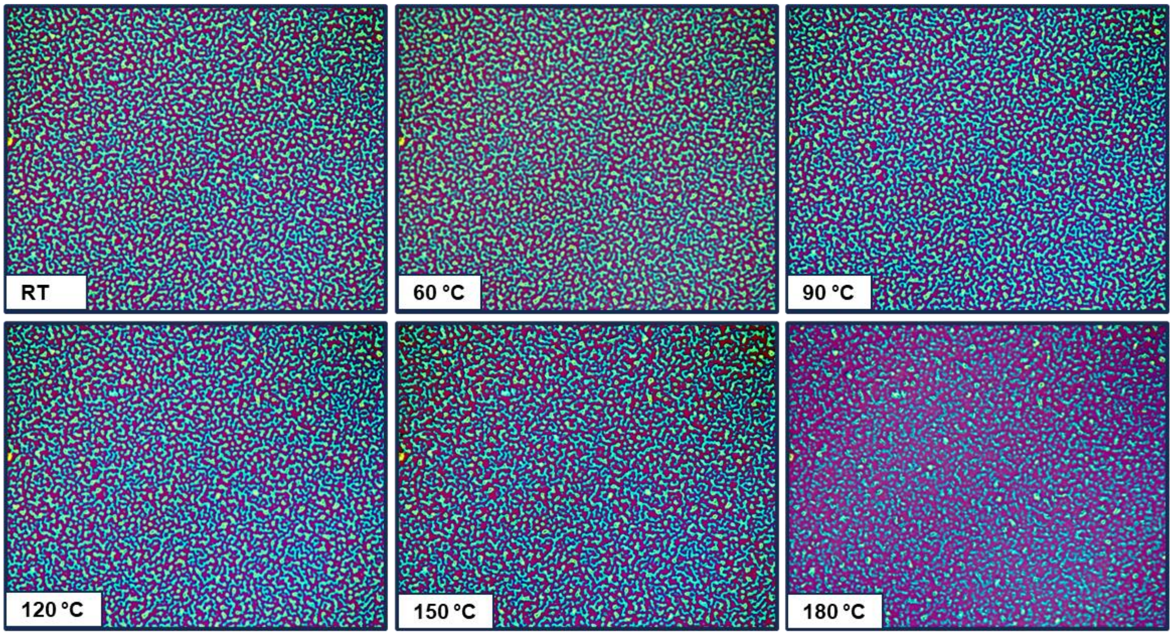

Figure S18. Comparison of optical micrographs of PUF structures subjected to sequential annealing from room temperature to 180°C in 30°C increments with 1-hour dwell periods.

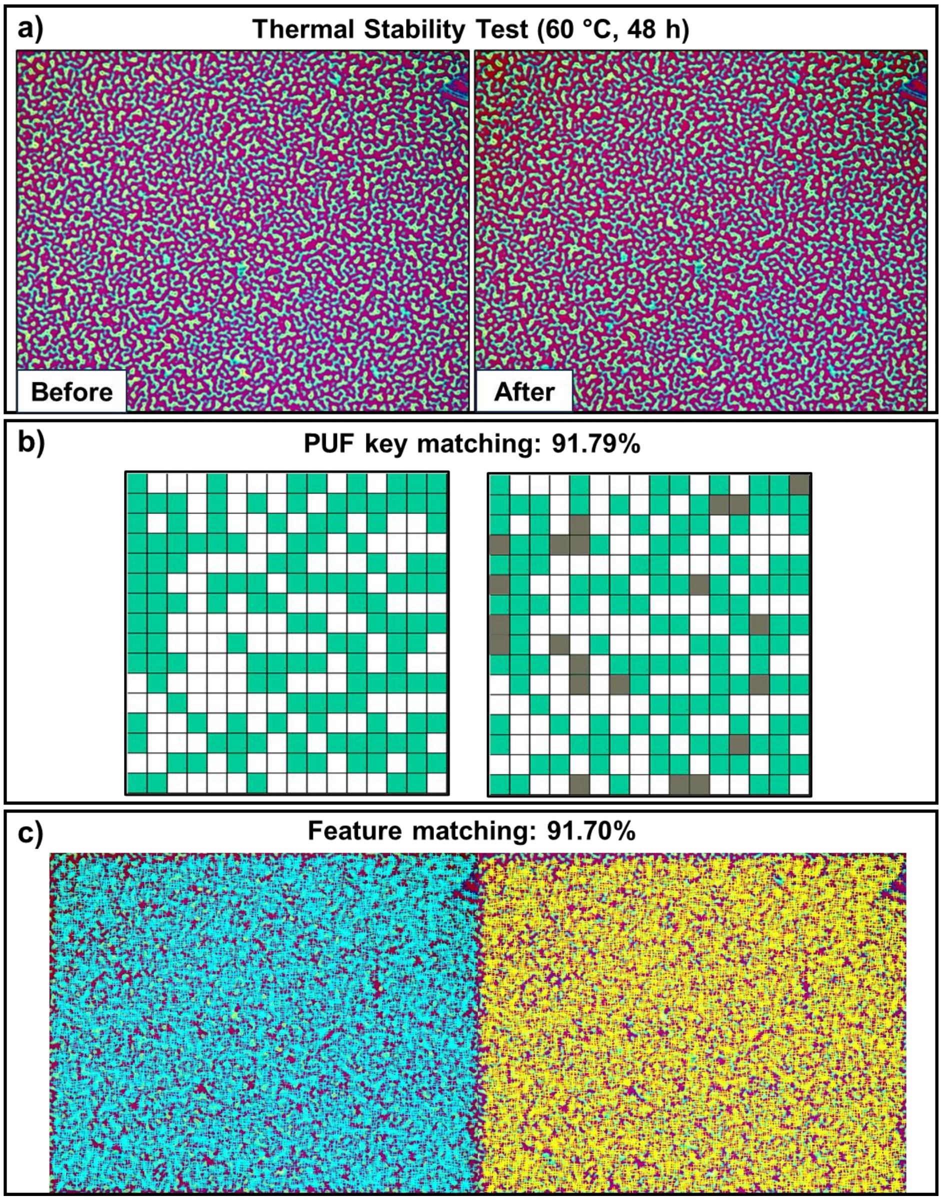

Figure S19. Thermal stability of the *molecular*-PUFs after 48 hours of continuous heating at 60 °C. a) Optical microscopy images before and after the thermal stability test. (b) Comparison of binary PUF keys extracted from the corresponding images and their similarity score. c) Direct authentication (and authentication score) through detection and matching of key points via a feature-matching algorithm.

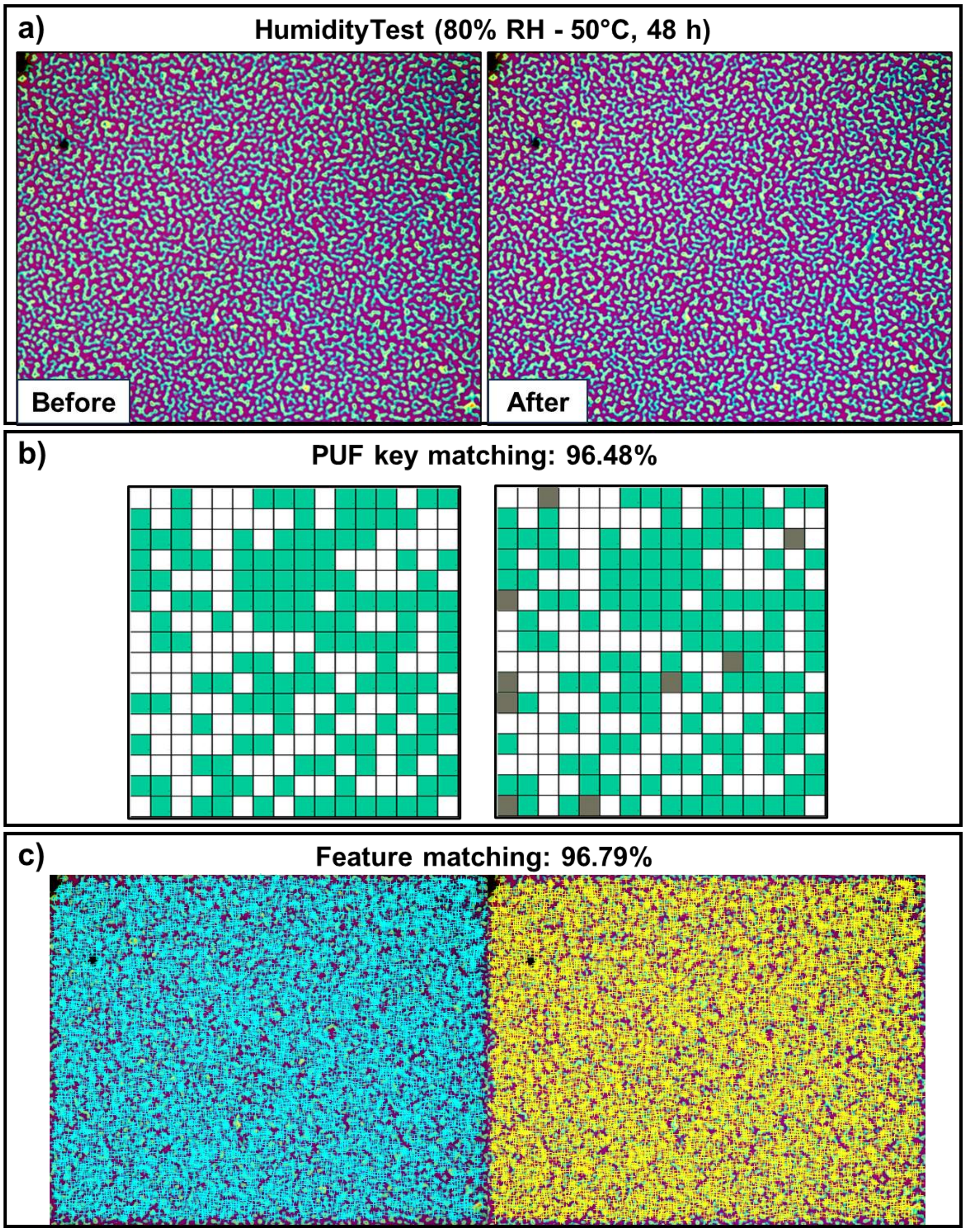

Figure S20. Stability of *molecular*-PUFs exposed to 80% relative humidity at 50°C for 48 hours. (a) Optical microscopy image of the PUF structure before and after the test. (b) Comparison of binary PUF keys extracted from the corresponding images and their similarity score. (c) Direct authentication (and authentication score) through detection and matching of key points via a feature-matching algorithm.

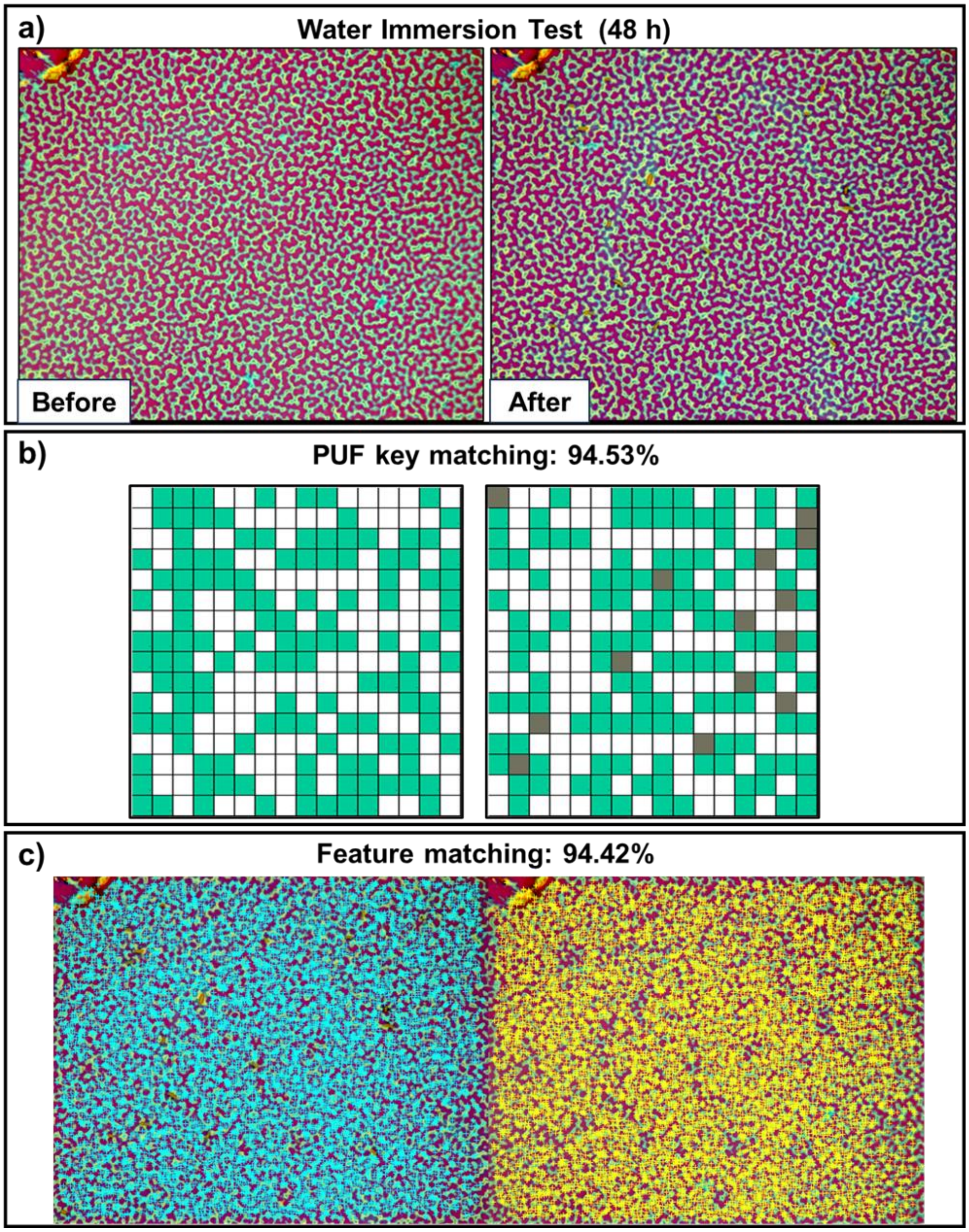

Figure S21. Stability of *molecular*-PUFs after 48 hours of water immersion. (a) Optical microscopy image of the PUF structure before and after the test. (b) Comparison of binary PUF keys extracted from the corresponding images and their similarity score. (c) Direct authentication (and authentication score) through detection and matching of key points via a feature-matching algorithm.

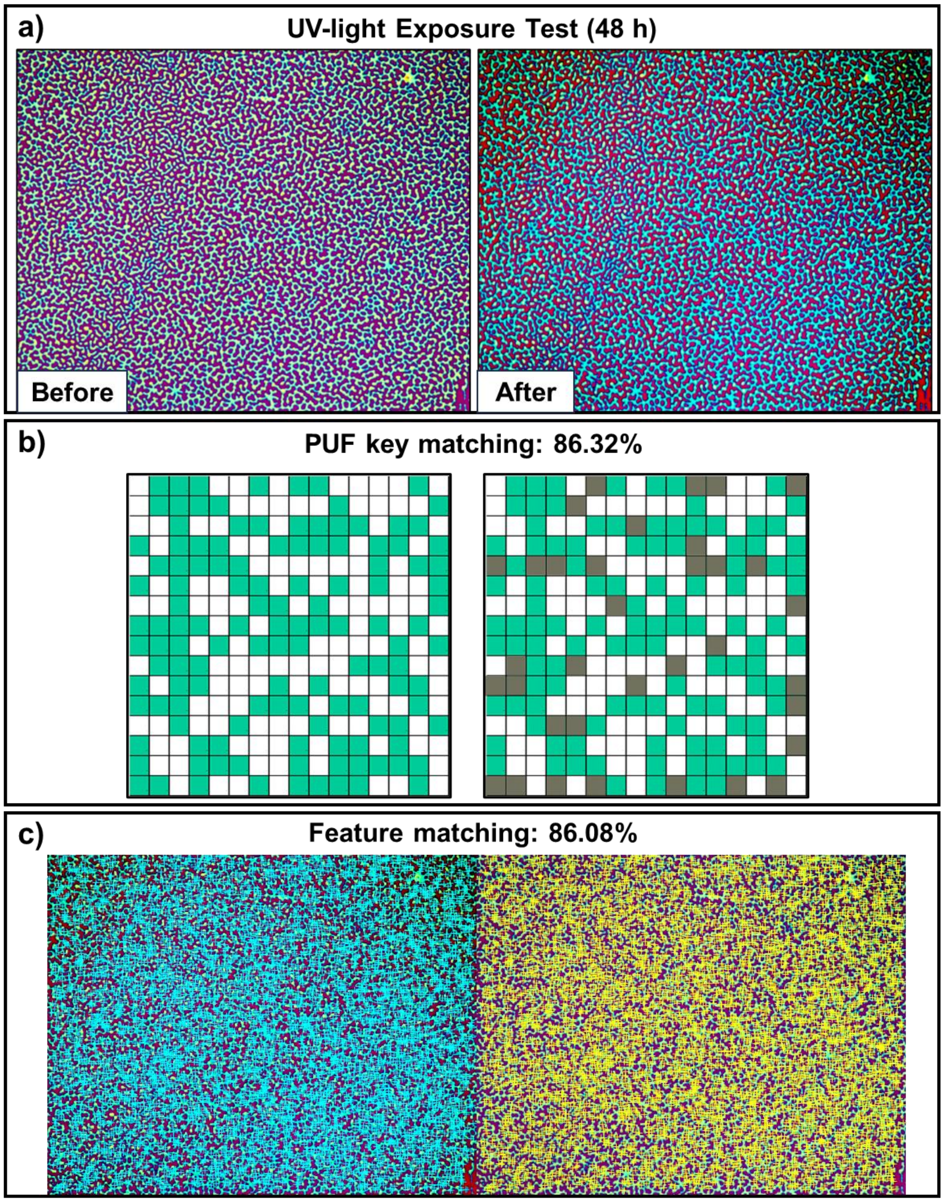

Figure S22. Stability of *molecular*-PUFs after 48 hours of UV illumination (365 nm, 10 W). (a) Optical microscopy image of the PUF structure before and after the test. (b) Comparison of binary PUF keys extracted from the corresponding images and their similarity score. (c) Direct authentication (and authentication score) through detection and matching of key points via a feature-matching algorithm.

References

(1) Bruker (**2014**) APEX2, version 2014.11-0, Bruker AXS Inc., Madison, Wisconsin, USA

(2) Bruker (**2013**) SAINT, version V8.34A, Bruker AXS Inc., Madison, Wisconsin, USA.

(3) Bruker (**2014**) SADABS, version 2014/4, Bruker AXS Inc., Madison, Wisconsin, USA.

(4) Bruker (**2010**) SHELXTL, version 6.14, Bruker AXS Inc., Madison, Wisconsin, USA.

(5) A. L. Spek, *Acta Crystallographica Section D: Biological Crystallography*, **2009**, 65, 148-155.

(6) C. F. Macrae, P. R. Edgington, P. McCabe, E. Pidcock, G. P. Shields, R. Taylor, M. Towler, J. van de Streek, *Journal of Applied Crystallography*, **2006**, 39, 453-457.

(7) Bae, H.J.; Bae, S.; Park, C.; Han, S.; Kim, J.; Kim, L.N.; Kim, K.; Song, S.-H.; Park, W.; Kwon, S. Biomimetic Microfingerprints for Anti-Counterfeiting Strategies. *Adv. Mater.* **2015**, 27, 2083-2089.

(8) Chen, G.; Weng, Y.; Wang, W.; Hong, D.; Zhou, X.; Wu, C.; Zhang, Y.; Yan, Q.; Yao, J.; Guo, T. Spontaneous Formation of Random Wrinkles by Atomic Layer Infiltration for Anticounterfeiting. *ACS Appl. Mater. Interfaces* **2021**, 13, 23, 27548-27556.

(9) Kim, K.; Kim, SU.; Choi, MY.; Saeed, M. H.; Kim, Y.; Na, JH. Voxelated Opto-Physically Unclonable Functions Via Irreplicable Wrinkles. *Light Sci. Appl.* **2023**, 12, 245.

(10) Ma, T.; Li, T.; Zhou, L.; Ma, X.; Yin, J.; Jieng, X. Dynamic Wrinkling Pattern Exhibiting Tunable Fluorescence for Anticounterfeiting Applications. *Nat. Commun.* **2020**, 11, 1811.

(11) Sun, N.; Chen, Z.; Wang, Y.; Wang, S.; Xie, Y.; Liu, Q. Random Fractal-Enabled Physical Unclonable Functions with Dynamic AI Authentication. *Nat. Commun.* **2023**, 14, 2185.

(12) J. H. Kim, S. Jeon, J. H. In, S. Nam, H. M. Jin, K. H. Han, G. G Yang, H. J Choi, K. M. Kim, J. Shin, S. W. Son, S. J Kwon, B. H. Kim, S. O. Kim, *Nat. Electron.* **2022**, 5(7), 433-442.

(13) Tian, L.; Liu, KK.; Fei, M.; Tadepalli, S.; Cao, S.; Gelmeier J.A.; Tsukruk V. V.; Singamaneni, S. Plasmonic Nanogels for Unclonable Optical Tagging. *ACS Appl. Mater. Interfaces* **2016**, 8, 6, 4031–4041.

(14) You, K.; Lin, J.; Wang, Z.; Jiang, Y.; Sun. J; Lin. Q.; Fu, H.; Guo, X.; Zhao, Y.; Lin, L.; Liu, Y.; Li, F. Biomimetic Fingerprint-like Unclonable Optical Anticounterfeiting System with Selectively In Situ-Synthesized Perovskite Quantum Dots Embedded in Spontaneous-Phase-Separated Polymers. *ACS Appl. Mater. Interfaces* **2025**, 17, 3, 5254–5267.

(15) Ma, M.; Jiang, Z.; Ma, T.; Gao, X.; Li, J.; Liu, M.; Yan, J.; Jiang, X. Robust PUF Label Authentication System Synergistically Constructed by Hierarchical Pattern of Self-Assembled Phase-Separation Encrypted Wrinkle and Deep Learning Model. *Adv. Funct. Mater*. **2024**, *34*, 2405239.
